# The role of the primary motor cortex in motor imagery: A theta burst stimulation study

**DOI:** 10.1111/psyp.14077

**Published:** 2022-05-03

**Authors:** Pamela Barhoun, Ian Fuelscher, Michael Do, Jason L. He, Andris Cerins, Soukayna Bekkali, George J. Youssef, Daniel Corp, Brendan P. Major, Dwayne Meaney, Peter G. Enticott, Christian Hyde

**Affiliations:** ^1^ Cognitive Neuroscience Unit, School of Psychology Deakin University Geelong Victoria Australia; ^2^ Department of Forensic and Neurodevelopmental Sciences, Sackler Institute for Translational Neurodevelopment Institute of Psychiatry, Psychology, and Neuroscience, King’s College London London UK; ^3^ Murdoch Children’s Research Institute, Centre for Adolescent Health Royal Children’s Hospital Melbourne Victoria Australia

**Keywords:** continuous theta burst stimulation, hand rotation task, internal modeling, motor imagery, primary motor cortex, typical motor development

## Abstract

While mentally simulated actions activate similar neural structures to overt movement, the role of the primary motor cortex (PMC) in motor imagery remains disputed. The aim of the study was to use continuous theta burst stimulation (cTBS) to modulate corticospinal activity to investigate the putative role of the PMC in implicit motor imagery in young adults with typical and atypical motor ability. A randomized, double blind, sham‐controlled, crossover, offline cTBS protocol was applied to 35 young adults. During three separate sessions, adults with typical and low motor ability (developmental coordination disorder [DCD]), received active cTBS to the PMC and supplementary motor area (SMA), and sham stimulation to either the PMC or SMA. Following stimulation, participants completed measures of motor imagery (i.e., hand rotation task) and visual imagery (i.e., letter number rotation task). Although active cTBS significantly reduced corticospinal excitability in adults with typical motor ability, neither task performance was altered following active cTBS to the PMC or SMA, compared to performance after sham cTBS. These results did not differ across motor status (i.e., typical motor ability and DCD). These findings are not consistent with our hypothesis that the PMC (and SMA) is directly involved in motor imagery. Instead, previous motor cortical activation observed during motor imagery may be an epiphenomenon of other neurophysiological processes and/or activity within brain regions involved in motor imagery. This study highlights the need to consider multi‐session theta burst stimulation application and its neural effects when probing the putative role of motor cortices in motor imagery.

## Abstract 2

A controlled continuous theta burst stimulation protocol was adopted to examine the role of the primary motor cortex in motor imagery. While corticospinal excitability was suppressed in individuals with typical motor ability, no changes in imagery performance were detected after applying active stimulation to the motor regions. This suggests that motor regions may not be causally implicated in motor imagery and/or that multiple stimulation sessions may be required when inducing cognitive‐behavioral changes.

## 1 INTRODUCTION

Mental simulation of action in the absence of overt movement is known as motor imagery (MI) (Decety, 1996a, 1996b; Guillot, Di Rienzo, et al., 2012; Lotze & Halsband, 2006). It is generally accepted that imagined movements provide insight into the internal representations that subserve voluntary movements (Jeannerod & Decety, 1995; Munzert et al., 2009). Consistent with this view, several studies have reported that mentally simulated actions display temporal congruence with real movement (Decety et al., 1989; Guillot, Hoyek, et al., 2012; Papaxanthis et al., 2002), equivalent speed‐accuracy trade‐offs (Cerritelli et al., 2000; Stevens, 2005; Ter Horst et al., 2010), and activate sensorimotor regions similarly to actual movement (Grosprêtre et al., 2016; Hardwick et al., 2017; Hétu et al., 2013). Given the proposed (relative) functional equivalence between MI and motor execution, there has been a growing interest in the potential of MI as a therapeutic tool for neurorehabilitation (Lotze & Cohen, 2006; Mulder, 2007). The logic here being that MI may facilitate activation of neural systems that support movement in instances where overt action may not be possible following injury (e.g., due to stroke) or delayed development. While evidence regarding the efficacy of MI interventions is promising, it nonetheless remains varied (Guerra et al., 2017; Mulder, 2007). Since MI represents a cost effective and accessible treatment adjunct to traditional therapies aimed at facilitating motor recovery and function, there is a clear need to better understand the factors contributing to these varied treatment outcomes. One compelling reason for the latter pertains to limitations in our understanding of the neural mechanisms underpinning MI (Hanakawa, 2016; Munzert et al., 2009), in particular the role of the primary motor cortex (PMC). Elucidating the neural basis of MI is therefore critical to the development and application of clinical interventions employing MI.

Recognized as the terminal site of neural impulses that comprise the motor commands which are transmitted to the peripheral nervous system, the PMC forms the principal brain area for the initiation of voluntary movement (Penfield & Boldrey, 1937; Stinear et al., 2009). While the functional importance of the PMC to overt movement is clear, evidence speaking to its involvement in mentally simulated actions is only partially supported by functional neuroimaging studies (Alkadhi et al., 2004; Dechent et al., 2004; Hanakawa et al., 2003, 2008; Richter et al., 2000). Some have suggested that the low temporal resolution and delayed blood oxygen level dependent (BOLD) response inherent to neuroimaging techniques may not be optimized for detecting whether the PMC is activated during MI functioning, and thus contributing to the heterogeneous findings (Hétu et al., 2013). While several approaches have been applied to determine what role, if any, the PMC plays in imagined movement, transcranial magnetic stimulation (TMS)‐based approaches that examine the effects of modulating the PMC on MI performance are arguably best suited to addressing this important issue (e.g., Aono et al., 2013; Facchini et al., 2002; Ganis et al., 2000; Roosink & Zijdewind, 2010; Tomasino et al., 2005; Williams et al., 2012). Repetitive pulses of TMS (rTMS) to the PMC can induce plastic changes in neural activity that persist beyond the point of administration (Karabanov et al., 2015). When applied to the hand node of the PMC, increases (i.e., long‐term potentiation [LTP]‐like) or decreases (i.e., long‐term depression [LTD]‐like) in excitability can be induced (Hoogendam et al., 2010; Maeda et al., 2000), as measured by motor evoked potentials (MEPs).

To date, a limited number of studies have employed rTMS to temporarily disrupt the PMC during performance of the hand rotation task (HRT), a well‐validated measure of implicit MI (Butson et al., 2014; Spruijt et al., 2015). Here, participants are instructed to discern the laterality of hand stimuli presented at varying angles and postural orientations. Individuals engaging in a MI strategy when completing the task generally demonstrate reduced efficiency (i.e., slower and less accurate) when responding to hand rotations with greater (i.e., lateral rotations) compared to lower (i.e., medial rotations) biomechanical complexity. As such, these biomechanical constraints displayed in HRT performance are thought to be unique to motoric forms of imagery (Butson et al., 2014; Spruijt et al., 2015). These studies have produced varied results, with one reporting that HRT performance was modulated following rTMS to the PMC (Pelgrims et al., 2011), while another did not (Cona et al., 2017). Importantly, participants in these studies either did not consistently engage in MI across all task stimuli (Pelgrims et al., 2011) or specifically self‐reported using a visual imagery (VI) strategy (Cona et al., 2017) instead of MI. Briefly, VI is a non‐motoric form of imagery for which involvement of the PMC would be expected to be negligible. Given that PMC (and broader motor) activity during HRT performance might be expected to be strongest when participants engage a MI strategy, including data from those participants who used alternative strategies (e.g., VI) would likely lead to an underestimation of any effect of rTMS to the motor cortices on MI performance.

Furthermore, neurophysiological response variability to rTMS protocols is well‐documented (Hamada et al., 2013; Hamada & Rothwell, 2016; López‐Alonso et al., 2014), with resultant changes in excitability reported in the expected direction, opposite direction, or even being absent. As a result of these earlier studies not reporting neurophysiological measures for rTMS responses, it is difficult to deduce whether previously reported mixed findings of PMC involvement in MI might be attributed to the inter‐individual variability in rTMS response that is commonly observed across participants (Hamada et al., 2013; Hamada & Rothwell, 2016; López‐Alonso et al., 2014). Taken together, the degree to which previous behavioral changes (or lack thereof) observed on the HRT following transient disruption to the PMC can be attributed to application of rTMS to motor regions, and/or engagement in MI performance, remains unclear and, by extension, so does the putative involvement of the PMC during MI.

In the last 15 years, however, an advanced patterned form of rTMS, continuous theta burst stimulation (cTBS), has provided an innovative alternative to traditional rTMS protocols (Huang et al., 2005). While cTBS protocols have also been associated with inter‐subject variability in stimulation response (e.g., Hamada et al., 2013; Huang et al., 2017; Jannati et al., 2017), cTBS requires less administration time (i.e., 40 s vs. ~10–45 min; Chung et al., 2015; Fitzgerald et al., 2006) and stimulus intensities to induce comparably long‐lasting inhibitory effects on brain activity (Goldsworthy et al., 2012; Huang et al., 2005; Karabanov et al., 2015; Lefaucheur et al., 2020) to the conventional rTMS paradigms adopted by earlier studies of PMC activity during MI. This improves tolerability for participants and reduces the risk of experimental error (e.g., subtle coil or head movement) that can hinder the longer rTMS procedures, such as those adopted by earlier work examining PMC involvement during HRT performance (Cona et al., 2017; Pelgrims et al., 2011). Consequently, cTBS may be an ideal form of noninvasive brain stimulation to explore the role of the PMC in implicit MI.

The primary aim of this study was to investigate the putative role of the PMC in implicit MI in young adults with typical motor ability. An additional subgroup of adults with atypical motor ability (i.e., Developmental Coordination Disorder [DCD]) were also included for further exploratory analysis to examine whether the findings differed across motor status, since research has shown that MI performance is less efficient in this group compared to individuals with typical motor skill, and that PMC excitability observed during HRT performance may alter as a function of motor ability (Hyde et al., 2014, 2017, 2018; Kashuk et al., 2017). Accordingly, a randomized, double‐blind, sham‐controlled, crossover, offline design was adopted in the current study. Across three separate sessions, participants received either active or sham cTBS to the PMC or supplementary motor area (SMA) prior to completing the HRT and letter number rotation task (LNRT). The SMA was targeted as an “active control” site, given previous work has more consistently implicated the SMA in both motor sequencing and mental rotation (Cona et al., 2017; Cona & Semenza, 2017). The LNRT, which engages a non‐motoric form of imagery (i.e., VI), was also included to determine if any observed effect on HRT performance following cTBS could be attributed to MI, as opposed to general rotation effects. Furthermore, individual performance profiles on the HRT for the sham condition were assessed to determine which participants were likely to engage in a MI strategy during the HRT (see method Section 2.4.2). Finally, MEP amplitudes induced by single‐pulse TMS were also recorded as a measure of whether cTBS had the expected inhibitory effect on PMC corticospinal excitability.

Based on the hypothesis that the PMC is causally involved in MI performance, it was predicted that participants who showed a tendency to adopt MI would demonstrate a reduction in HRT performance when PMC activity was interrupted using cTBS. Assuming that the PMC is not substantively involved in non‐motoric cognitive processes, no change in performance on the LNRT was expected following application of cTBS. It was further hypothesized that participants would show a reduction in both HRT and LNRT performance when SMA activity was disrupted, given research demonstrates that the SMA is important for object rotation and imagery (Cona et al., 2017; Cona & Semenza, 2017). Finally, it was predicted that these effects would not differ according to motor status (i.e., individuals with typical motor ability and DCD), with the exception of PMC involvement during MI. Specifically, unlike healthy controls (Hyde et al., 2017), prior research shows that PMC excitability is absent during MI use in individuals with DCD (Hyde et al., 2018), which may indicate that the PMC is not active (or activity is attenuated) during MI proficiency in this disorder. It was thus hypothesized that participants with DCD would not demonstrate the same reduction in HRT performance when PMC activity was downregulated using cTBS.

## 2 METHOD

### 2.1 Participants

The sample comprised 35 young adults aged 18 to 27 years. Demographic information for participants is presented in Table 1. Analysis showed no difference in sex and age between groups (see Supporting Information A). All participants self‐reported being right handed (*n* = 33) or were ambidextrous showing a right hand preference (*n*_Control_ = 2) on the Edinburgh Handedness Inventory [revised] (Williams, 1986). The project was advertised on Australian university websites and social media channels (e.g., Facebook and Gumtree), directed at young adults with and without motor difficulties. All participants provided written informed consent and were screened for TMS contraindicators. The project received ethical clearance from Deakin University Human Research Ethics Committee (DUHREC).

**TABLE 1:** Participant demographic information

Group	Sample size	Age		Sex (N^0^)		Mean BOT‐2 (%tile)
		Mean	Range (years)	Male	Female	
Control	25	22.12 (2.55)	19–27	12	13	24.36th (15.54th)
DCD	10	22.00 (2.90)	18–26	1	9	10.20th (4.37th)

*Note*: Standard deviation presented in brackets.

Abbreviations: BOT‐2, Bruininks‐Oseretsky test of motor proficiency, Second Edition (measure of motor proficiency); DCD, developmental coordination disorder.

While the majority of participants completed all three sessions in the current study (please see Section 2.3 for counterbalancing information), a portion of participants only completed one (*n*_Control_ = 5; *n*_DCD_ = 2) or two sessions (*n*_Control_ = 1). Accordingly, 29 participants (*n*_Control_ = 21; *n*_DCD_ = 8) received “active PMC” stimulation, 29 participants received “active SMA” stimulation (*n*_Control_ = 20; *n*_DCD_ = 9) and 32 participants received sham stimulation (*n*_Control_ = 23; *n*_DCD_ = 9) (see Sections 2.2 and 2.3).

Participants with typical motor ability presented with age‐appropriate motor proficiency as indicated by motor ability above the 18th percentile on the Bruininks‐Oseretsky Test of Motor proficiency, Second Edition (BOT‐2: Bruininks, 2005), a well validated and reliable measure for detecting motor impairments in young adults (Hands et al., 2015; McIntyre et al., 2017). All participants with DCD were selected according to DSM‐5 criteria and recent guidelines for identifying DCD in adults (Barnett et al., 2015). Briefly, these participants had BOT‐2 scores at or below the 16th percentile (*n*_0–5th_ = 1; *n*_6th–10th_ = 5; *n*_11th–16th_ = 4), indicating that their motor ability was significantly below that expected for their age. Participants also self‐reported having difficulties with movement in childhood and adulthood as indicated by the well‐validated Adult Dyspraxia/Developmental Coordination Disorder Checklist (ADC: Kirby et al., 2010) or during consultation (for more detail regarding DCD selection criteria see Barhoun et al., 2021; Hyde et al., 2014, 2018). No participants reported having any other neurodevelopmental disorders (e.g., ADHD or Dyslexia) or medical and neurological conditions (e.g., cerebral palsy). All participants presented with intelligence within the normal range, as determined by a total score at or above 80 (i.e., “low average”) on the Wechsler Abbreviated Scale of Intelligence—Second Edition (WASI‐II: Wechsler, 2011), or because they were undertaking, or previously had completed, an undergraduate degree at university.

### 2.2 Measures

#### 2.2.1 Noninvasive brain stimulation

##### Single‐pulse transcranial magnetic stimulation (TMS)

**FIGURE 1:**
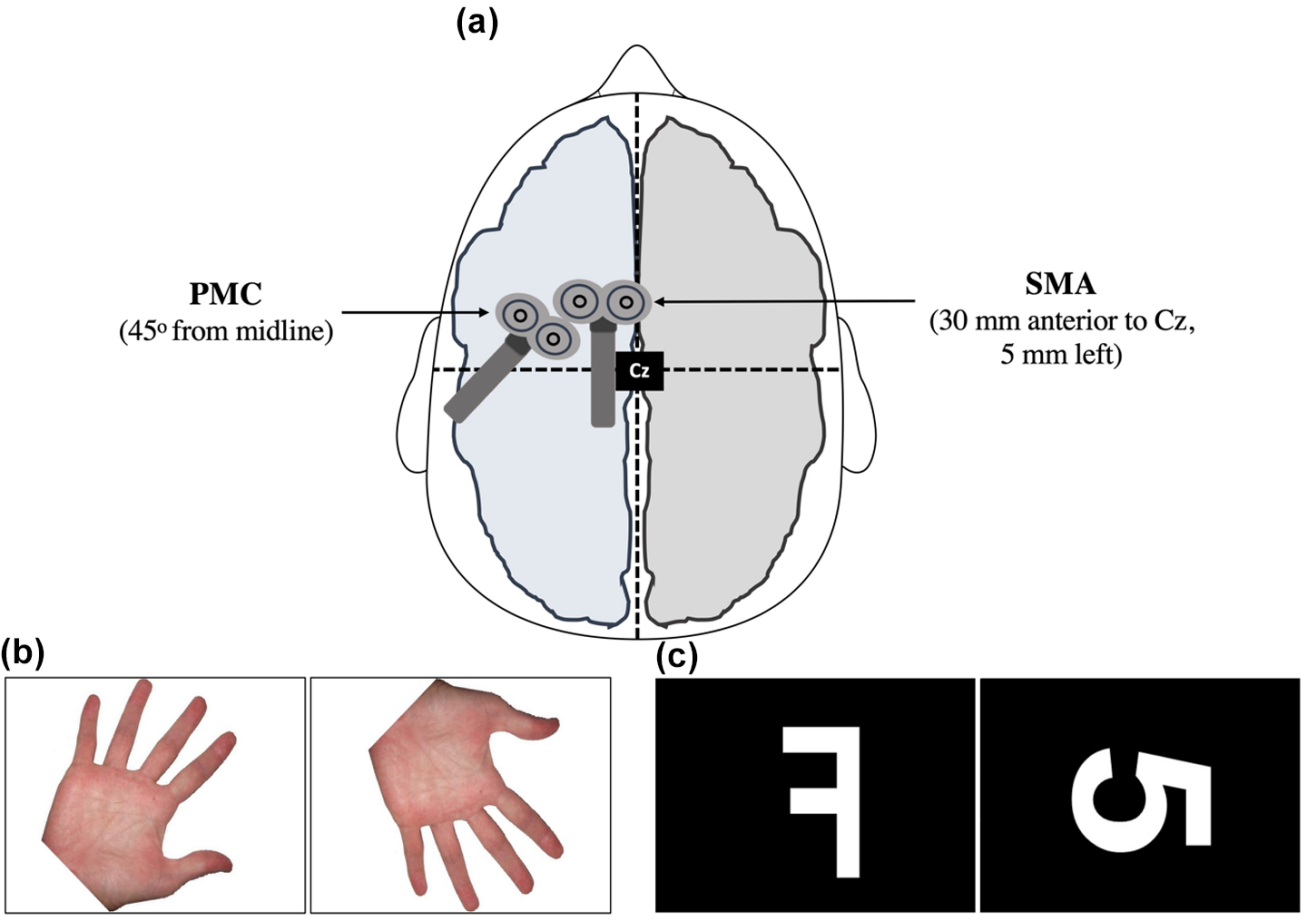
Coil positions and target brain regions for brain stimulation, and stimuli used for rotation tasks. PMC, primary motor cortex; SMA, supplementary motor area; (a) PMC and SMA coil positions for single pulse and cTBS stimulation. TMS coils are not to scale; (b) HRT (hand rotation task): right‐hand stimulus rotated 45° (lateral) and left‐hand stimulus rotated 135° (medial); (c) LNRT (letter number rotation task): “flipped” letter F stimulus at 0° and “unflipped” letter 5 stimulus rotated 90°

Single‐pulse TMS producing a monophasic pulse was delivered with a Magstim‐200 stimulator via a figure‐of‐eight handheld coil (70 mm) (Magstim Company Ltd, UK) and was applied over the PMC in the left hemisphere by placing the coil against the scalp with the handle pointing posterior to the head and positioned 45° away from the midline (see Figure 1a). Motor evoked potentials (MEPs) elicited by the right first dorsal interosseous muscle (FDI) were recorded using disposable self‐adhesive electromyography (EMG) electrodes (10 mm). The reference and ground electrodes were placed 2 cm anterior along the right first index finger and on the right ulnar styloid process, respectively. EMG activity was amplified (x1000) with a BioAmp (AD Instruments, USA) using a Powerlab 4/35 system and digitized (10 kHz) and recorded using LabChart version 8 software (AD Instruments, USA).

Single‐pulse TMS was administered to establish the site over the PMC that produced a test stimulus (TS) with an average peak to peak MEP amplitude of 1 mV across 20 consecutive trials in the right FDI muscle and was recorded prior to the application of theta burst stimulation to provide a baseline measurement of corticospinal activity. This measurement block (20 trials) was later repeated at 5 min and 15 min after applying theta burst stimulation to assess any change in corticospinal activity (see Section 2.3).

Measurements of corticospinal activity from a subset of participants (*n* = 17) were originally obtained using a figure‐eight handheld coil (70 mm) with the Magstim Rapid^2^ (Magstim Company Ltd, UK), which produces a biphasic pulse. However, many participants required a stimulation intensity greater than 80% of maximum stimulator output (MSO) to achieve an average MEP of 1 mV, which exceeded our laboratory’s safety guidelines and led to a high attrition rate. Therefore, the remainder of participants (*n* = 18) had these measurements recorded using the aforementioned Magstim 200 stimulator that delivers a monophasic pulse and required a lower % MSO to produce the required MEP amplitude of 1 mV. Analysis found no significant differences between MEP amplitudes using the monophasic or biphasic pulse (see Supporting Information B).

##### Continuous theta burst stimulation (cTBS)

cTBS (Magstim Rapid^2^, Magstim Company Ltd, UK) was applied using a figure‐of‐eight air film cooled coil (70 mm) that was supported by a proprietary mechanical stand. cTBS was administered over the PMC in the left hemisphere, using the same coil position and orientation as the single pulses described above (see Section 2.2.1.1). Consistent with previous studies (Cona et al., 2017; Hamada et al., 2008; Janssen et al., 2015), cTBS was also administered over the SMA in the left hemisphere located 30 mm anterior to the vertex in the sagittal midline of the scalp (i.e., Cz according to the international 10–20 EEG system) and 5 mm left, with the coil positioned posteriorly to the head (see Figure 1a). We must acknowledge the possibility that cortices adjacent to the SMA were stimulated. However, several studies using both TMS and neuronavigational systems have shown the SMA is likely to be stimulated when applying TMS 30 mm anterior to the cortex location for the tibialis anterior (TA) muscle (near Cz) (Terao et al., 2005, 2007). As such, we can be confident that the SMA, or a portion thereof, was stimulated by cTBS in our study.

The cTBS protocol was applied at 70% resting motor threshold (RMT) and consisted of three pulses delivered at 50 Hz repeated every 200 ms (i.e., 5 Hz) for 40 s, which yielded a total of 600 pulses. RMT for cTBS was defined as peak to peak MEP amplitudes greater than .05 mV across at least five of ten consecutive trials, and was established after baseline using a figure‐eight handheld coil (70 mm) with the Magstim Rapid^2^ (Magstim Company Ltd, UK). Sham cTBS was applied to either the PMC or SMA (see Section 2.3) using the same aforementioned cTBS protocol and with an identical figure‐of‐eight air film cooled placebo coil (70 mm) that produces similar sound and sensation but does not interfere with neural activity. Accordingly, the application of sham cTBS (including PMC/SMA location, coil orientation, and position) was administered using an identical application method as outlined above for active cTBS.

#### 2.2.2 Rotation tasks

MI and VI ability were measured with the HRT and LNRT, respectively, programmed using the E‐prime software package (Version 2.0, Psychology Software Tools, Pittsburgh, PA, USA). The stimuli (see Sections 2.2.2.1 and 2.2.2.2) were centered in the middle of a 15‐inch Acer computer screen presented randomly at 45° increments between 0° and 360°. A fixation cross appeared at the center of the screen for 1000 ms prior to each stimulus, while the test stimuli remained on the screen for a maximum of 10 s or until a response was detected. Participant reaction time (RT) was recorded to the nearest 1 ms via the E‐prime software package once a response (including accuracy) had been provided by pressing the designated response keys on the computer keyboard (see Section 2.3).

##### Hand rotation task

The stimuli were high resolution pictures of a left or right hand (9 cm by 8 cm; see Figure 1b) shown only in palm‐view orientation (i.e., palm facing toward the participants). Participants completed five practice trials (or were repeated until they were comfortable completing the task), followed by 40 randomly administered test trials (split across left‐ and right‐hand stimuli). Performance across all stimuli was combined across angular rotations of 0°, 45°, 90°, 135°, and 180°, resulting in eight test trials per angle. Participants were not given specific instructions prompting MI.

##### Letter number rotation task

The stimuli were high resolution images of either the letter F or the number 5 (9 cm by 6 cm; see Figure 1c). Half of the images were mirror reversed (i.e., “flipped”) while the other half were facing the correct direction (i.e., how they are normally written; “unflipped”). Participants completed five practice trials (or were repeated until they were comfortable completing the task), followed by 40 randomly administered test trials (split across the letter F and number 5 stimuli). Performance across all stimuli was combined across angular rotations of 0°, 45°, 90°, 135°, and 180°, yielding eight test trials per angle.

### 2.3 Procedure

Participants attended three separate sessions, the order of which was counterbalanced across subjects using an automated algorithm. In two of the sessions, participants received active cTBS to the PMC (“active PMC”) or SMA (“active SMA”). In the other session, they received sham cTBS to either the PMC or SMA, with the site of stimulation counterbalanced across participants. Accordingly, this study was designed so that all participants would have received active stimulation to both the PMC and SMA, and sham stimulation to either the PMC or SMA, after completing their participation in the study. While the initial randomization of these sessions was counterbalanced to be equally distributed across subjects, as a result of a portion of participants being unable to complete all sessions and attrition, not all possible condition orders were completed equally. Both the participant and researcher administering the stimulation were blinded to each condition (i.e., active or sham) (see Supporting Information C for the blinding procedure adopted during testing) and were asked to report whether they thought active or sham stimulation was being administered after completing the session (see Supporting Information D). It is also important to note that in order to minimize the potential for participant burden and reduce the likelihood of attrition, the current study implemented one sham session (split between sham PMC and sham SMA) in line with previous work (Cona et al., 2017), instead of using two sham sessions, one for each stimulation target (i.e., PMC and SMA). Although unlikely, in instances where the stimulation target was the same across two consecutive sessions (e.g., session 1 = “active PMC,” session 2 = sham PMC, and session 3 = “active SMA”), it may have been possible for a participant to deduce that the location stimulated twice had an active and sham condition, and therefore inferred that the third session would be an active condition. However, no significant difference was detected in the proportion of participants that reported being either correct (i.e., predicted the correct condition), incorrect (i.e., predicted the wrong condition), or unsure (i.e., stated being unsure of the condition type) of the stimulation condition for each condition type (i.e., “active PMC,” “active SMA,” or sham PMC/SMA), *χ* ^2^ (4, 62) = 7.21, *p* = .125 (see Supporting Information D). Still, future research applying TBS protocols to investigate the role of different brain regions in MI may want to consider adopting a sham session for each stimulation target of interest.

**FIGURE 2:**
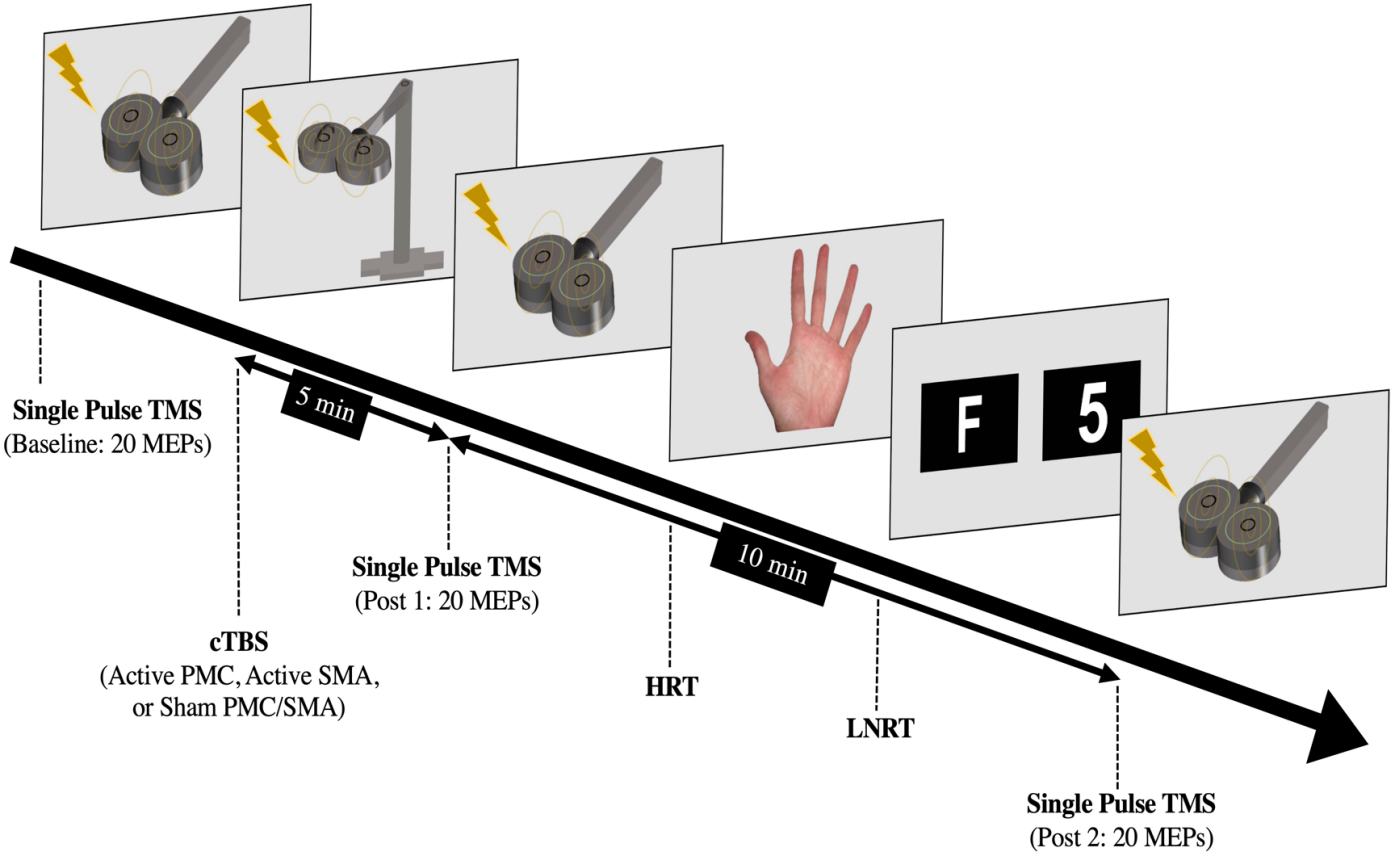
Experimental procedure across all sessions. PMC, primary motor cortex; SMA, supplementary motor area; The order of HRT (hand rotation task) and LNRT (letter number rotation task) administration was randomized across sessions for each participant

Across all sessions prior to cTBS application, 20 MEPs were recorded for baseline measurement of corticospinal activity by administering single‐pulse TMS to the PMC. Subsequently, cTBS (either active or sham) was applied to the relevant area (either PMC or SMA). Participants were then asked to sit motionless for 5 min in order to obtain the full effect of cTBS (Huang et al., 2005). Immediately following, 20 MEPs at the 1 mV TS were recorded (i.e., post‐cTBS 5 min) by re‐applying single‐pulse TMS to the PMC. Participants then completed the HRT and LNRT, which were counterbalanced across all three sessions across participants. Similar to above, while the initial randomization of these tasks were counterbalanced to be equally distributed across sessions and subjects, due to a portion of participants not completing all three sessions and attrition, not all task orders across sessions were completed equally. For each task, participants sat at a comfortable distance in front of a screen, with both hands resting palm down on the appropriate response keys of a computer keyboard that was placed on a desk in front of the participant. Before each trial, participants were asked to fixate on the cross at the center of the screen until the target stimulus appeared. For the HRT, participants were instructed to determine whether the picture was a left or right hand as quickly and accurately as possible, using the two designated keys (“d” for left‐hand stimuli and “k” for right‐hand stimuli). For the LNRT, participants were instructed to determine whether pictures of a letter F or number 5 were “unflipped” (i.e., facing the correct direction) or “flipped” (i.e., mirror reversed) as quickly and accurately as possible, by also pressing the designated keys (“d” for “unflipped” stimuli and “k” for “flipped” stimuli). Completion of both tasks was achieved within 10 min to ensure the effect of cTBS had not passed (Huang et al., 2005). Finally, 20 MEPs were then recorded (i.e., post‐cTBS 15 min) by readministering single‐pulse TMS to the PMC (see Figure 2).

### 2.4 Design and analysis

**FIGURE 3:**
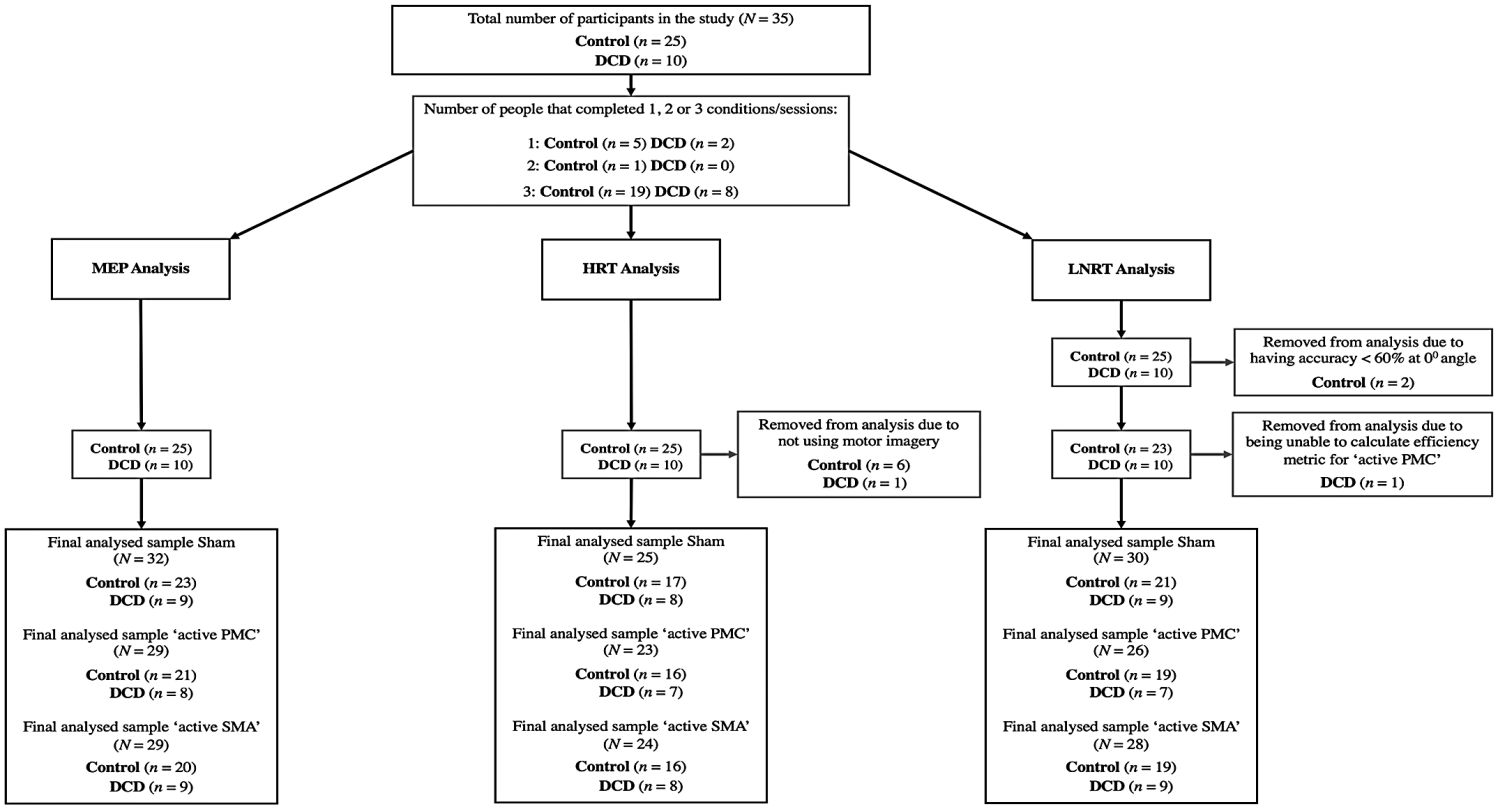
Sample size flow diagram for MEP, HRT, and LNRT analyses. DCD, developmental coordination disorder; HRT, hand rotation task; LNRT, letter number rotation task; MEP, motor evoked potential

All analyses were primarily conducted using the “rprime” (Mahr, 2020), “tidyverse,” “lme4,” “ggplot2,” and “sjPlot” packages (Bates et al., 2014; Lüdecke et al., 2021; Wickham, 2011; Wickham et al., 2019) in R Core Team (2013). Cohen’s *d*_*z*_ effect size values were calculated using the MOTE Effect Size Calculator from the DOOM lab (Buchanan et al., 2017). See Figure 3 for the final sample size used for each of the main analyses described below. Observed power calculations are also available in Supporting Information E.

#### 2.4.1 MEPs

To assess changes in excitability following cTBS, separate linear mixed models using restricted maximum likelihood estimation (REML) were conducted for each condition (i.e., sham, “active PMC,” “active SMA”), with MEP amplitude at each trial as the dependent variable, and with MEP time point (pre‐cTBS vs. post‐cTBS 5 min and post‐cTBS 15 min) and MEP time point x group (control vs. DCD) as fixed effects. All models contained a random intercept to account for clustering of MEP amplitudes in each condition within individuals, and subject variability at baseline.

#### 2.4.2 MI and VI performance

Mean RT and accuracy (proportion of correct responses across trials) for each angle in both tasks were calculated for each participant in each condition, and averaged across angular rotation (i.e., 0°, 45°, 90°, 135°, and 180°). Correct and incorrect trials were used in the analysis. Premature responses were removed by excluding trials with RT less than 250 ms (*n*_HRT_“active SMA” trials_ = 1; *n*_LNRT_“active PMC“ trials_ = 1). Any trials without a response were also removed (*n*_HRT_sham trials_ = 1). Consistent with our previous work (Barhoun et al., 2021; Fuelscher, Williams, Enticott, et al., 2015; Hyde et al., 2017, 2018 ), mean efficiency was used as the primary measure of performance on both tasks (higher scores = reduced MI ability), and was calculated for each participant by dividing mean RT by mean accuracy at each of the stimuli presentation conditions (see Hyde et al., 2014 for a detailed discussion regarding the efficiency measure). Briefly, our own research group has consistently adopted the efficiency measure to better characterize HRT performance in children (Fuelscher, Williams, Hyde, 2015; 2016) and adults (Barhoun et al., 2021; Hyde et al., 2014, 2017) with both typical motor ability and DCD. Assuming that appropriate statistical assumptions are fulfilled (see Supporting Information F for relevant efficiency metric assumptions), we have observed that incorporating RT and accuracy into a single metric (as per mean efficiency) can provide a more sensitive and reliable measure of HRT performance than RT or accuracy used alone, particularly when assessing individuals with DCD (Barhoun et al., 2019).

While the HRT implicitly elicits a MI strategy in most individuals, previous studies have shown that a small proportion (20%–30%) of participants typically implement non‐motoric strategies to complete the task (Hyde et al., 2017; Mibu et al., 2020). Accordingly, we identified participants who were likely to have engaged in MI (as per Hyde et al., 2017, 2018) by examining individual performance profiles on the HRT for the sham condition to confirm whether they were consistent with the biomechanical constraints of movement, which are unique to MI (Butson et al., 2014; Spruijt et al., 2015). This was inferred when performance was more efficient for biomechanically simpler rotations (i.e., medial) compared to challenging rotations (i.e., lateral) by any absolute mean efficiency value (i.e., MI = lateral efficiency value > medial efficiency value) (Hyde et al., 2017, 2018). Medial rotations were calculated as the average efficiency across responses for left‐hand stimuli presented at 45°, 90°, and 135° and right‐hand stimuli presented at 225°, 270°, and 315°. Conversely, lateral rotations were computed as the average responses for left‐hand stimuli presented at 225°, 270°, and 315° and right‐hand stimuli presented at 45°, 90°, and 135°. As a result, 19 of 25 individuals with typical motor ability and 9 of 10 individuals with DCD were likely to have used MI, and therefore included in any subsequent analyses of HRT performance. Three participants, however, did not complete a sham condition as they were unable to attend all three sessions. Therefore, we are unable to confirm whether the HRT performance profiles of these individuals are consistent with the biomechanical constraints of movement as per MI. Further analysis found no significant difference in MI performance across conditions when excluding these participants (see Supporting Information G), and thus they were included in the present analyses.

Finally, to ensure that participants could discriminate between left‐ and right‐hand stimuli, accuracy across angular rotation of 0° for both the HRT and LNRT was assessed to ensure participants were able to complete the tasks above chance level (i.e., 60% ~ 5 or more correct trials presented at 0°) (Hyde et al., 2017, 2018). All participants met this requirement, with the exception of two participants from the control group when completing the LNRT and they were removed from the following analyses. One participant in the DCD group was also unable to complete all trials correctly across angular rotations of 180° for the LNRT for the PMC condition, and therefore an efficiency measure could not be calculated. Performance from this condition was removed from subsequent LNRT analyses.

To compare mental rotation performance (i.e., MI and VI) across conditions, separate linear mixed models using REML were conducted for both the HRT and LNRT, with performance efficiency as the dependent variable and with condition (sham vs. “active PMC” and “active SMA”) and condition x group (control vs. DCD) as fixed effects. Both models contained a random intercept to account for clustering of efficiency scores in each condition within individuals. Further exploratory analysis was conducted to examine if MEP change (pre‐cTBS vs. post‐cTBS 5 min and post‐cTBS 15 min) was correlated with HRT efficiency (see Supporting Information H).

### 2.5 Data and code availability

In accordance with DUHREC ethics approval and the consent provided by participants, group level data that support the findings of the current study are available upon reasonable request from the corresponding author [P.B]. The raw data used in this study are not publicly available due to ethical restrictions. Analyses for this study was conducted using an open source software (R Core Team, 2013). The code script for key analyses conducted are publicly available on the Open Science Framework: https://osf.io/6rzym/.

## 3 RESULTS

### 3.1 Analyses of MEP data

**FIGURE 4:**
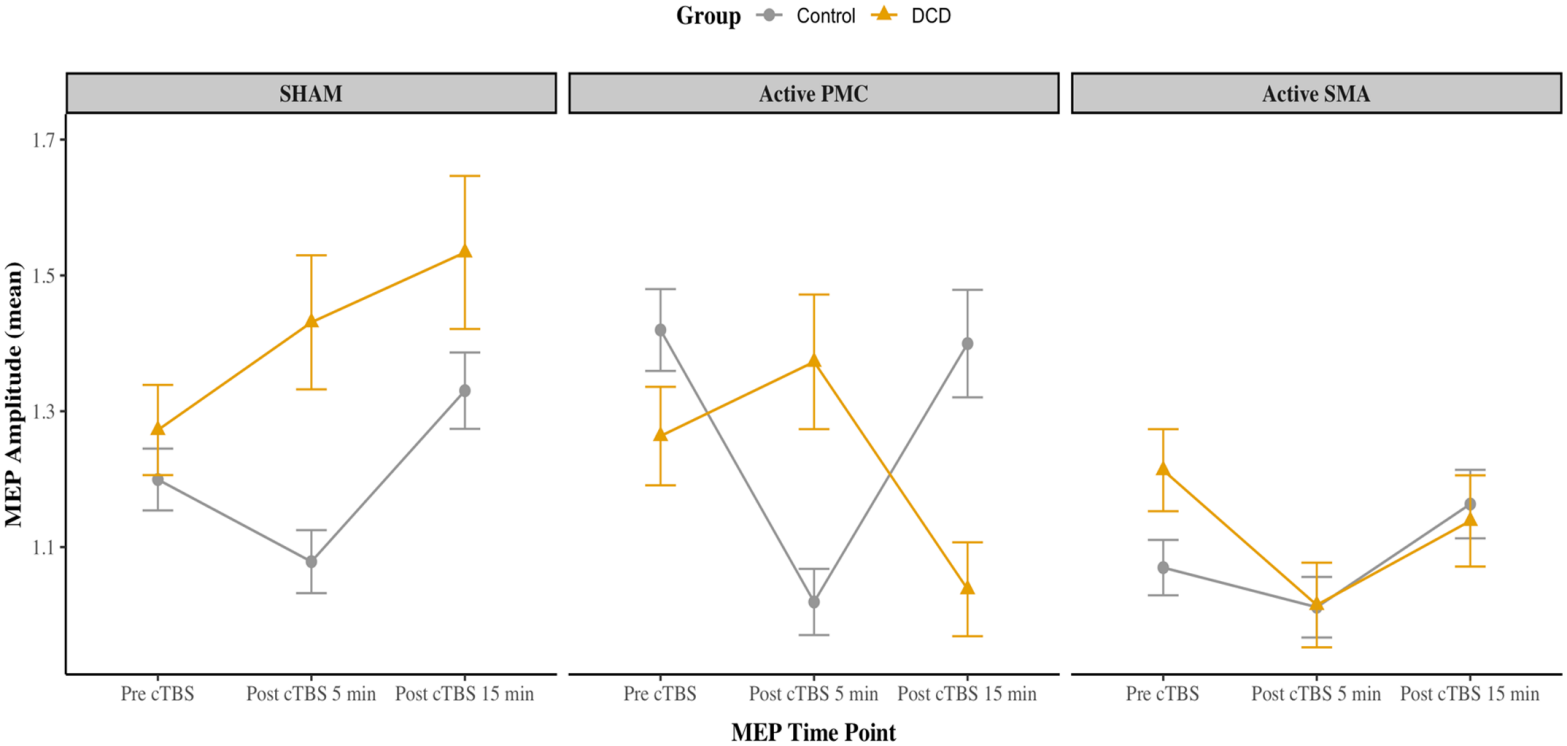
MEP amplitudes across MEP time point and condition. Standard error presented; DCD, developmental coordination disorder; MEP, motor evoked potential; PMC, primary motor cortex; SMA, supplementary motor area

Descriptive statistics for MEP time point are presented in Table 2 and modeled in Figure 4 (see also Supporting Information I for individual data points for participants with DCD).

**TABLE 2:** Means and standard errors for MEP amplitudes for each condition

Condition	Sample size	MEP time point		
		Pre‐cTBS	Post‐cTBS 5 min	Post‐cTBS 15 min
SHAM				
Control	23	1.20 (0.05)	1.08 (0.05)	1.33 (0.06)
DCD	9	1.27 (0.07)	1.43 (0.10)	1.53 (0.11)
Active PMC				
Control	21	1.42 (0.06)	1.02 (0.05)	1.40 (0.08)
DCD	8	1.26 (0.07)	1.37 (0.10)	1.04 (0.07)
Active SMA				
Control	20	1.07 (0.04)	1.01 (0.04)	1.16 (0.05)
DCD	9	1.21 (0.06)	1.01 (0.06)	1.14 (0.07)

*Note*: Standard error presented in brackets.

Abbreviations: DCD, developmental coordination disorder; MEP, motor evoked potential; cTBS, continuous theta burst stimulation; PMC, primary motor cortex; SMA, supplementary motor area.

The linear mixed models revealed a statistically significant main effect for MEP time point for “active PMC” (*F* [2, 1709] = 8.19, *p* < .001) and sham conditions (*F* [2, 1886] = 7.62, *p* = .001), however, there were significant interactions between MEP time point and group for both conditions (see Supporting Information J). Exploration of these interaction effects (see Table 3) suggested that there was a significant large reduction in MEP amplitudes following “active PMC” stimulation at post‐cTBS 5 min compared to pre‐cTBS for individuals with typical motor skill, however, this reduction was not observed for the DCD group. No reduction was detected at post‐cTBS 5 min for either group for the sham condition. Furthermore, a significant moderate increase in MEP amplitudes was revealed following sham stimulation at post‐cTBS 15 min compared to pre‐cTBS for the DCD group but failed to reach significance for the typically developing group. These effects were not observed for the “active PMC” condition.

**TABLE 3:** Exploration of interaction effects for MEP time point for controls and individuals with DCD

	Test statistics					Effect size		
	Estimates (B)	95% Confidence Interval		*t*	*p*	*d*_*z*_	95% Confidence Interval	
		Lower	Upper				Lower	Upper
Active PMC								
Control								
Post‐cTBS 5 min versus Pre‐cTBS	−0.40 (0.08)	−0.55	−0.25	−5.20	**<.001**	−1.09	−1.63	−0.54
Post‐cTBS 15 min versus Pre‐cTBS	−0.02 (0.08)	−0.17	0.13	−0.26	.795	−0.05	−0.48	0.37
DCD								
Post‐cTBS 5 min versus Pre‐cTBS	0.11 (0.13)	−0.14	0.35	0.88	.381	0.30	−0.42	1.00
Post‐cTBS 15 min versus Pre‐cTBS	−0.23 (0.13)	−0.47	0.02	−1.81	.070	−0.63	−1.37	0.16
SHAM								
Control								
Post‐cTBS 5 min versus Pre‐cTBS	−0.12 (0.07)	−0.25	0.01	−1.80	.071	−0.36	−0.78	0.07
Post‐cTBS 15 min versus Pre‐cTBS	0.13 (0.07)	−0.00	0.26	1.95	.051	−0.39	−0.81	0.04
DCD								
Post‐cTBS 5 min versus Pre‐cTBS	0.16 (0.11)	−0.05	0.47	1.48	.139	0.48	−0.22	1.17
Post‐cTBS 15 min versus Pre‐cTBS	0.26 (0.11)	0.05	0.47	2.44	**.015**	0.79	0.01	0.51

*Note*: Standard error presented in brackets.

Significance of bold values in *p* < .05.

Abbreviations: cTBS, continuous theta burst stimulation; DCD, developmental coordination disorder; PMC, primary motor cortex.

The linear mixed model for “active SMA” stimulation revealed a statistically significant main effect for MEP time point, *F* (2, 1709) = 4.79, *p* = .008. Specifically, a small reduction at post‐cTBS 5 min compared to pre‐cTBS across both groups was detected, *B* = −0.10, *SE* = 0.05, 95% CI [−0.20, −0.01], *t* (1709) = −2.14, *p* = .032, *d*_*z*_ = −0.37, 95% CI_*dz*_ [−0.74, 0.01]. No change was observed at post‐cTBS 15 min and no interactions between MEP time point and group were observed (see Supporting Information J).

### 3.2 Analyses of MI and VI data

**FIGURE 5:**
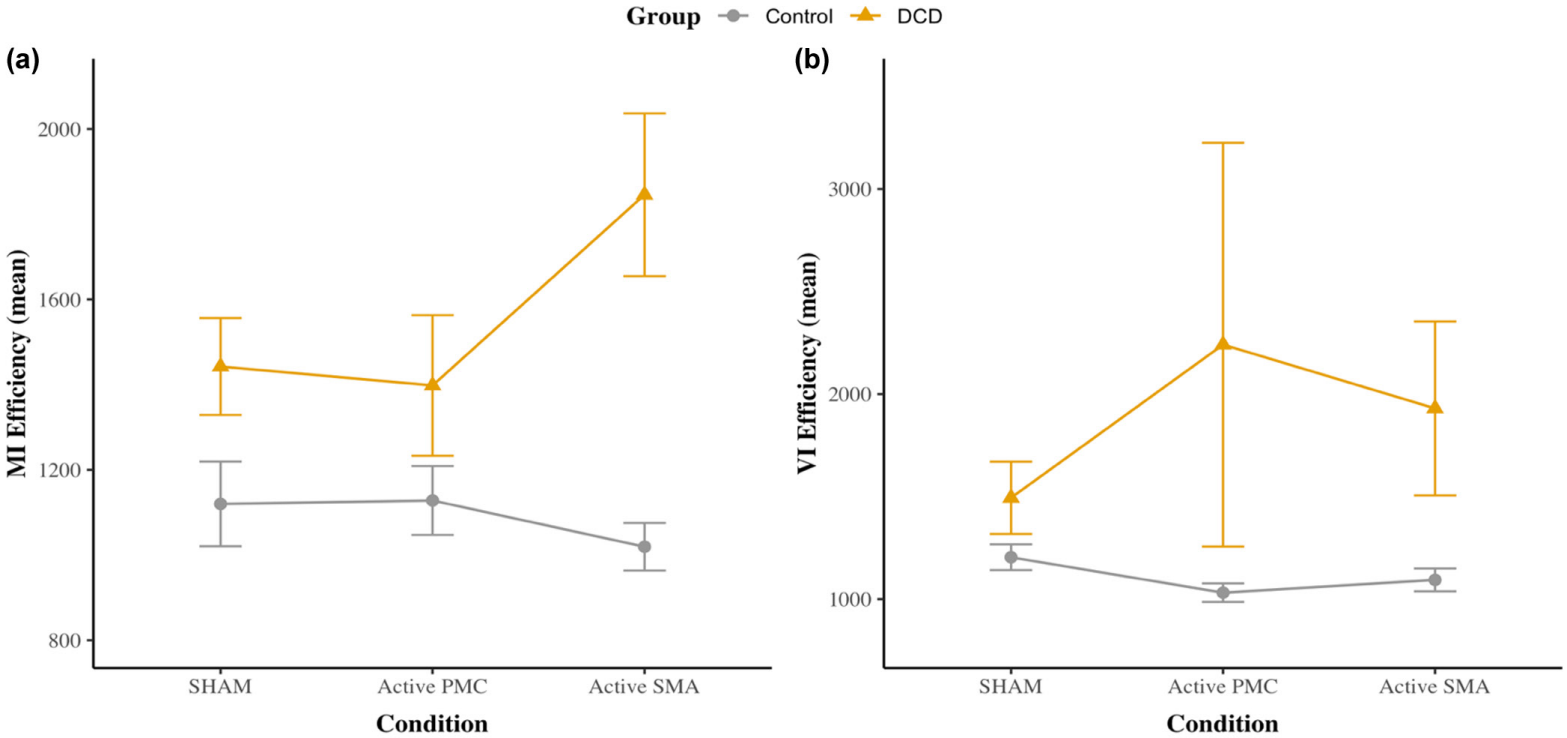
MI and VI performance for controls and individuals with DCD across conditions. Standard errors presented; DCD, developmental coordination disorder; MI, motor imagery; VI, visual imagery; (a). MI efficiency as indicated by performance on the hand rotation task; (b) VI efficiency as indicated by letter number rotation task performance

Descriptive statistics for MI and VI performance are presented in Table 4 and modeled in Figure 5. See also Supporting Information K for these descriptive statistics using RT and accuracy metrics, as well as Supporting Information L for a discussion on possible order effects.

**TABLE 4:** Mean efficiency and standard error for motor and visual imagery performance across conditions

Condition	HRT (ms)		LNRT (ms)	
	Sample size		Sample size	
SHAM				
Control	17	1120.09 (99.32)	21	1204.75 (62.83)
DCD	8	1442.57 (113.69)	9	1494.66 (175.99)
Active PMC				
Control	16	1128.14 (80.83)	19	1032.00 (45.19)
DCD	7	1398.15 (164.96)	7	2241.30 (984.49)
Active SMA				
Control	16	1019.51 (55.82)	19	1094.33 (55.69)
DCD	8	1845.78 (190.89)	9	1930.37 (424.07)

*Note*: Standard error presented in brackets.

Abbreviations: DCD, developmental coordination disorder; HRT, hand rotation task (measure of motor imagery); LNRT, letter number rotation task (measure of visual imagery); PMC, primary motor cortex; SMA, supplementary motor area.

The linear mixed models revealed no statistically significant main effect for condition for both MI (i.e., HRT efficiency; *F* [2, 339.18] = 0.64, *p* = .530) and VI performance (i.e., LNRT efficiency; *F* [2, 407.77] = 0.20, *p* = .821). Specifically, no statistically significant effect was detected for “active PMC” (HRT: *B* = 49.43, *SE* = 92.54, 95% CI [−131.94, 230.80], *t* (339.13) = 0.53, *p* = .593, *d*_*z*_ = .10, 95% CI_*dz*_ [−0.27, 0.46]; LNRT: *B* = 144.53, *SE* = 232.84, 95% CI [−311.83, 600.90], *t* (409.22) = 0.62, *p* = .535, *d*_*z*_ = 0.12, 95% CI_*dz*_ [−0.27, 0.51]) or “active SMA” conditions (HRT: *B* = 104.08, *SE* = 92.28, 95% CI [−76.78, 284.95], *t* (341.80) = 1.13, *p* = .259, *d*_*z*_ = 0.21, 95% CI_*dz*_ [−0.16, 0.58]; LNRT: *B* = 86.72, *SE* = 228.51, 95% CI [−361.16.10, 534.59], *t* (410.57) = 0.379, *p* = .705, *d*_*z*_ = 0.07, 95% CI_*dz*_ [−0.30, 0.44]). This indicated that across all individuals with typical motor ability and DCD, no significant changes in HRT and LNRT efficiency were detected following active stimulation to either the PMC or SMA, when compared to the sham condition (see Supporting Information M).

**FIGURE 6:**
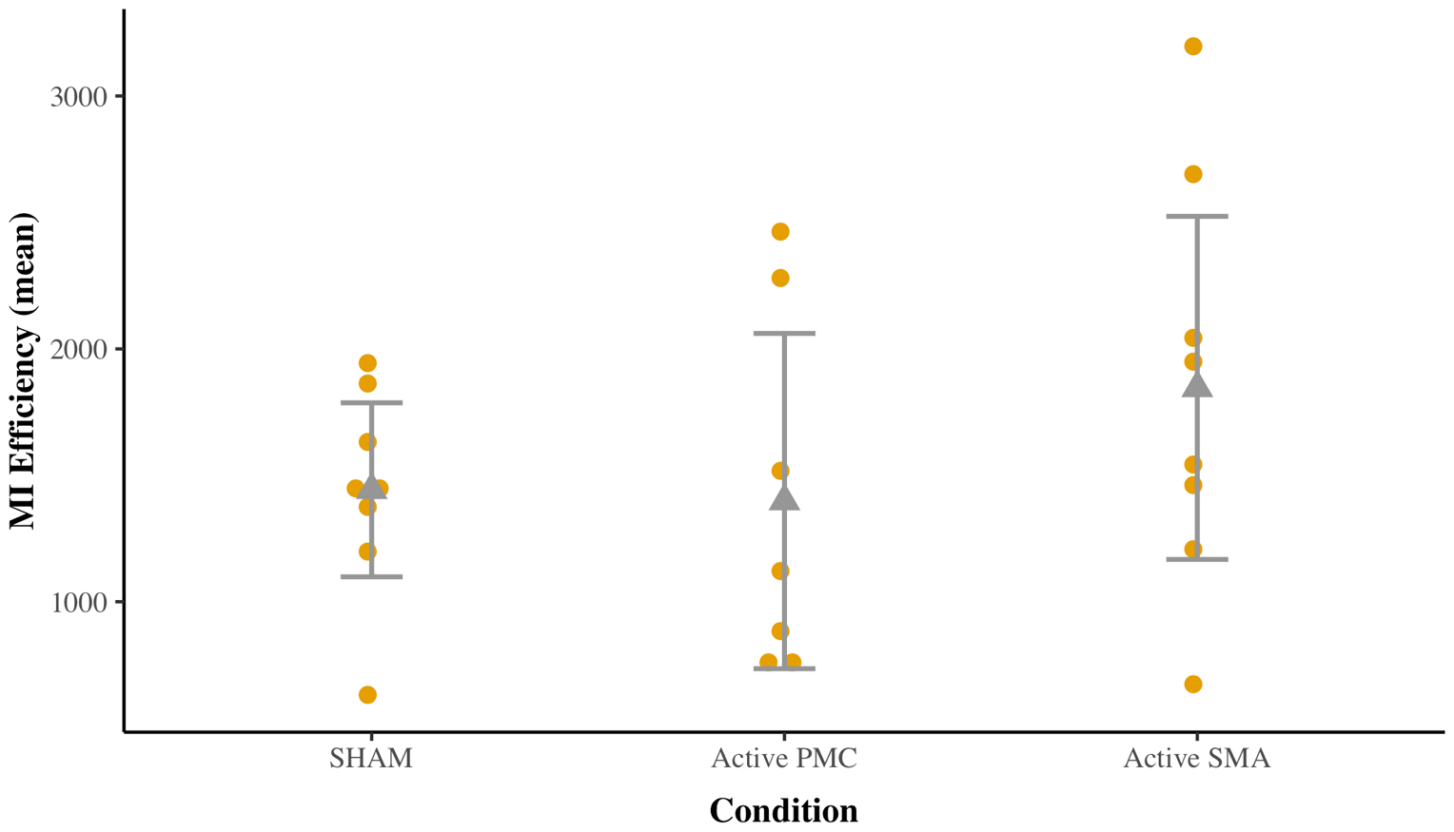
Individual data points for MI efficiency for individuals with DCD across conditions. Sample means and lower and upper 95% confidence intervals presented; MI efficiency as indicated by performance on the hand rotation task

While the overall test for a significant interaction between condition and group was not statistically significant for VI performance, *F* (2, 406.80) = 1.93, *p* = .147, it appeared to approach significance for MI performance, *F* (2, 338.05) = 3.00, *p* = .051. Exploration of this possible interaction revealed a significant increase in HRT efficiency for “active SMA” compared to sham for the DCD group, *B* = 360.04, *SE* = 162.00, 95% CI [40.60, 679.00], *t* (345) = 2.22, *p* = .027, *d*_*z*_ = 0.79, 95% CI_*dz*_ [−0.04, 1.57], but not for those with typical motor ability, *B* = −21.90, *SE* = 111.00, 95% CI [−241.20, 197.00], *t* (341) = −0.20, *p* = .844, *d*_*z*_ = −0.05, 95% CI_*dz*_ [−0.54, 0.44]. However, due to a limited number of participants in the DCD group for the “active SMA” condition (*n* = 8) and the large confidence interval, we urge caution in over interpreting this effect (see Figure 6 and Supporting Information N). Further inspection of the individual data points revealed that only 50% (4/8) of participants with DCD matched theoretical expectation (i.e., showed an increase in HRT efficiency), while the remainder of participants did not—this spread at the individual data point level was also substantial. As a result, while there may be a slight overall increase in mean MI performance for “active SMA” compared to sham for the DCD group, we are unable to infer to what extent this is a consistent pattern (Grice et al., 2020). Accordingly, on the basis of the above exploration, we chose not to interpret the initial interaction (*p* = .051) as evidence of a true effect.

## 4 DISCUSSION

The aim of the study was to examine the role of the PMC in implicit MI use in young adults with typical and atypical motor ability. A subgroup of adults with low motor skill (i.e., DCD) was also included to examine if the observed effects differed across motor status. Consistent with previous findings, our results showed that active cTBS had an inhibitory effect on PMC activity at post‐cTBS 5 min for those with typical motor development. While this effect was not observed in those with DCD, a nonsignificant trend toward a reduction in MEP amplitudes was detected at post‐cTBS 15 min, which may indicate a possible delayed or atypical response to cTBS application in people with motor difficulties. No such effects were observed following sham stimulation. While active cTBS to the PMC resulted in decreased corticospinal excitability, neither HRT nor LNRT performance were altered following active cTBS conditions (PMC or SMA) relative to sham cTBS. This effect did not differ across motor status (i.e., typical motor ability and DCD). These findings were not consistent with our hypothesis that the PMC would be directly involved in MI ability, and may suggest that the PMC (and SMA) are not causally implicated in MI. However, our study design and findings also highlight the possible limitations of applying single session cTBS protocols to motor regions to induce cognitive‐behavioral changes. These outcomes are discussed in detail below.

### 4.1 cTBS has an inhibitory effect on individuals with typical motor skill

Despite the reported putative inhibitory effects of cTBS (e.g., Goldsworthy et al., 2012; Huang et al., 2005; Karabanov et al., 2015), individual variability in cTBS response still remains common; ranging from inhibition or null effects, to facilitation (Do et al., 2018; Goldsworthy et al., 2014; Hamada et al., 2013; Jannati et al., 2017). However, our ability to infer what role (if any) motor regions play in MI using a noninvasive “virtual lesion” approach, such as cTBS, is predicted on the latter inducing the expected reduction in PMC excitability. Therefore, unlike earlier rTMS accounts of the role of the PMC in MI (Cona et al., 2017; Pelgrims et al., 2011), we sought to obtain a proxy measure (via MEPs) for PMC excitability pre‐ and post‐cTBS to ensure that the anticipated group level inhibitory effect was observed. Using MEPs to gauge cTBS induced alterations in excitability is consistent with previous work investigating the variability in cTBS response following stimulation of the PMC (e.g., Huang et al., 2005; Laviolette et al., 2013; Vernet et al., 2013). MEP amplitudes before (i.e., pre‐cTBS) and after cTBS application (i.e., post‐cTBS 5 min and post‐cTBS 15 min) were therefore recorded using single‐pulse TMS to provide a proxy measure for whether cTBS had an inhibitory effect on PMC excitability. While MEPs are used to indirectly measure modulation of corticospinal excitability in the absence of any other direct measures of cortical plasticity (e.g., electroencephalography; Vernet et al., 2013), cTBS applied to the PMC has nonetheless been shown to reduce MEP amplitudes as elicited by single‐pulse TMS in contralateral hand muscles, which is thought to indicate LTD‐like changes in corticospinal activity (Huang et al., 2005).

The current study found a significant reduction in MEP amplitudes following active cTBS applied to the PMC at post‐cTBS 5 min compared to baseline (i.e., pre‐cTBS), suggesting that cTBS had an inhibitory effect on PMC activity (viz corticospinal excitability). However, group contrasts revealed that this effect was only present in adults with typical motor ability and was not detected in individuals with DCD. In contrast, participants with DCD showed no reduction in MEP amplitude at post‐cTBS 5 min but displayed a discernible reduction in MEP amplitude at post‐cTBS 15 min (*d*_*z*_ = −0.63), albeit a nonsignificant decrease. Although we acknowledge this may be a result of the small sample size of adults with DCD, this trend towards a reduction in MEP amplitudes may also indicate a possible delay and/or atypical response to cTBS in people with low motor ability. While we are unable to confirm this response in the absence of any other TBS studies in DCD, other disorders affecting movement (e.g., autism spectrum disorder and progressive supranuclear palsy) have also presented atypical responses (e.g., showing excessive/longer lasting LTD‐like changes post‐cTBS or facilitatory cTBS aftereffects in MEPs rather than inhibitory) in cortical excitability following cTBS application to the PMC (Conte et al., 2012; Desarkar et al., 2021; Jannati et al., 2020). Nonetheless, the current decrease in corticospinal activity observed in individuals with typical motor ability (*d*_*z*_ = −1.09) appears to be consistent with earlier seminal research that found a moderate to large reduction in MEP amplitudes 5–30 min following cTBS application to the PMC (i.e., Cohen’s d = −.90 and –42.2% to –45.0%; Chung et al., 2016; Huang et al., 2005). As expected, no such effects were observed following sham stimulation at post‐cTBS 5 min compared to pre‐cTBS for either group, indicating that sham cTBS did not modulate neural activity. Therefore, given active cTBS had the expected inhibitory effect on PMC excitability, we can be confident that the absence of behavioral changes observed on the HRT and LNRT (see Section 4.2) following cTBS cannot be attributed to our rTMS approach having a null or unpredicted effect on neural activity.

Similar to PMC stimulation, the results revealed a small significant decrease in MEP amplitudes after SMA stimulation at post‐cTBS 5 min compared to pre‐cTBS in both groups. While we cannot be completely certain that cTBS was having an inhibitory effect on the SMA given these MEP amplitudes were induced by single‐pulse TMS to the hand node of the PMC, this evident reduction in corticospinal excitability may reflect a wider inhibitory network effect between the SMA and PMC. Indeed, the SMA (and broader premotor regions) are known to have direct anatomical and functional projections with the hand area of the PMC (Dum & Strick, 1991; Nachev et al., 2008). Research has shown that cTBS applied to the SMA may lead to modulation of the corticospinal pathway and induces changes in PMC excitability (Hamada et al., 2009; Laviolette et al., 2013). Likewise, our results are compatible with the idea that cTBS effectively stimulated the SMA, leading to modulation of PMC activity that is reflected by reduced MEP amplitudes at post‐cTBS 5 min.

Lastly, a significant increase in MEP amplitudes was observed after sham stimulation at post‐cTBS 15 min compared to pre‐cTBS for the DCD group (*d*_*z*_ = 0.79). While failing to reach significance, a discernible facilitation in corticospinal excitability was also detected for the control group at post‐cTBS 15 min following sham cTBS (*d*_*z*_ = 0.48). Given that sham cTBS did not appear to alter neural activity at post‐cTBS 5 min for either group, it is possible that the physical hand movement required to perform the tasks (i.e., HRT and LNRT) prior to this measurement may have led to an increase in motor excitability at post‐cTBS 15 min (Abbruzzese et al., 1996; Chen et al., 1998; Leocani et al., 2000), as opposed to pre‐cTBS where participants remained stationary. It should be noted, however, that while a significant effect was detected for participants with DCD at post‐cTBS 15 min following sham stimulation, further inspection of the individual data points shows large variability in MEP amplitudes at post‐cTBS 15 min for the sham condition for this group (see Supporting Information I). As a result, while an overall significant increase in MEP amplitudes at post‐cTBS 15 min following sham cTBS may have been detected, we are unable to reliably conclude whether this a true effect for individuals with DCD.

### 4.2 The role of motor regions in MI

Our results were not consistent with our hypothesis that the PMC would be directly involved in MI. Contrary to expectations, individuals who showed a tendency to adopt MI when completing the HRT did not show a significant reduction in performance efficiency after cTBS application to the PMC or SMA, relative to their HRT performance following sham cTBS. Since this study excluded participants who did not display HRT performance profiles that were consistent with the use of a MI strategy, we can therefore be confident that these observed findings on the HRT can be attributed to MI performance as opposed to general rotation ability. These null effects did not differ across motor status (i.e., typical motor ability and DCD) and were also observed for the LNRT. In support, further exploratory analyses (please see Supporting Information H) found no significant relationships between MEP change across time point and HRT performance across individuals for either the PMC or SMA. Thus, the results show that irrespective of whether active cTBS or sham stimulation was applied to the PMC or SMA, no changes in imagery performance were produced, despite evidence of cTBS‐induced neurophysiological modulation (i.e., reduction of MEPs). Although these findings have been unable to demonstrate that motor regions (i.e., PMC and SMA) are casually involved in implicit MI (or VI), there may be several possible explanations for this outcome.

First, it may be that neither the PMC nor SMA are directly or causally involved in imagery performance. Given that the PMC is largely responsible for regulating and executing voluntary movements, some authors have speculated that the PMC may not be involved in MI since this process does not necessitate the movement execution phase that the PMC is responsible for (Bhattacharjee et al., 2020; Penfield & Boldrey, 1937; Stinear et al., 2009). Furthermore, the majority of previous support for the involvement of both the PMC and SMA in imagery ability stems from correlational techniques, such as fMRI and single‐pulse TMS (e.g., Fulford et al., 2018; Guillot et al., 2009; Hétu et al., 2013; Hyde et al., 2017; Williams et al., 2012), which showed increases in PMC activity during mental imagery in the form of blood oxygen level dependent (BOLD) response or corticospinal excitability. However, such activity may have been the result of the PMC and SMA receiving input from other cortical (e.g., frontal, parietal and temporal areas) and subcortical (e.g., basal ganglia and cerebellum) regions thought to be important for internally generated movements and object perception (Ganis et al., 2004; Hardwick et al., 2017; Kraeutner, 2019; Zvyagintsev et al., 2013), rather than these motor regions being directly involved in mental imagery. In this respect, motor cortical activation may be an epiphenomenon of other processes involved in MI. Indeed, many of these areas have anatomical and/or structural projections to the motor cortices (for different reviews see Bhattacharjee et al., 2020; Nachev et al., 2008) and have been found to subserve imagery ability (Ganis et al., 2004; Guillot et al., 2009; Hardwick et al., 2017; Zvyagintsev et al., 2013). Accordingly, as an individual engages in imagery performance, it may be that these other brain regions are predominantly activated, leading to spill over activity into the motor areas that is being detected by earlier accounts.

Second, a single session application of cTBS to motor regions may not be sufficient for inducing observable cognitive‐behavioral effects (e.g., MI performance), even where these regions are causally involved and there is evidence of neurophysiological modulation. To the best of our knowledge, a limited number of studies have applied cTBS to the PMC and/or SMA (e.g., Derosiere et al., 2015, 2019; Solopchuk et al., 2017) in a single session to probe the neural basis of a given cognitive‐behavioral system. These studies failed to detect changes in performance following cTBS application or only observed subtle changes. While this lack of observed or consistent effect may reflect a true null, it may also raise the question as to whether single‐session application of cTBS to motor regions is sufficient to facilitate the type of plastic neural changes necessary to influence behavioral or cognitive performance. In support, evidence from recent clinical guidelines using rTMS and TBS protocols shows that repetitive stimulation (minimum of approximately three sessions) is required to achieve therapeutic success when addressing a range of psychological and/or behavioral outcomes (e.g., depression, pain perception, motor function; Lefaucheur et al., 2020). While this work pertains to clinical outcomes as opposed to cognitive‐behavioral effects, this report nonetheless highlights that single session cTBS may be inadequate for producing consistent cognitive‐behavioral changes, regardless of whether PMC excitability is altered.

Lastly, our data also highlight the possibility that the inhibitory effects induced by cTBS may not have persisted long enough for the reduction in neural excitability in the PMC and SMA to have impacted task performance. Indeed, while cTBS had an inhibitory effect on PMC activity at post‐cTBS 5 min in those with typical motor ability, this was not detected at post‐cTBS 15 min. Thus, the observed inhibitory effect on corticospinal excitability may have dissipated between these two time points (i.e., post‐cTBS 5 min and post‐cTBS 15 min), during which time the tasks were being administered. That is, it is possible that applying cTBS to the PMC may have inhibited MI ability (or VI ability) from the time of application till post‐cTBS 5 min, but that this effect did not persist long enough to impact HRT (or LNRT) performance being recorded between the post‐cTBS 5 min and post‐cTBS 15 min MEP acquisition time points. Consequently, the duration of induced corticospinal inhibition may not have been sufficient to produce changes in behavioral performance. Furthermore, there is some evidence to suggest that muscle activation may alter cTBS outcomes (Goldsworthy et al., 2014). Given that participants in the current study performed finger movements between post‐cTBS 5 min and post‐cTBS 15 min, we cannot rule out the possibility that such movement (though minimal) may have altered LTD‐like effects of cTBS on the PMC.

### 4.3 Implications and directions for future research

Taken together, the current study provides important insight into the role of motor regions in MI. In contrast to earlier work, this study was the first to determine whether HRT performance profiles were consistent with a MI strategy prior to including participants in any analyses, and further provided a measure of corticospinal excitability following cTBS application to confirm the group level effect of rTMS on PMC activity. Accordingly, by adopting a controlled and innovative study of HRT performance using an advanced “transient lesion” approach to TMS application, this work offers some important evidence that the PMC (and SMA) may not be directly involved in MI performance. Instead, previously detected PMC activation during HRT performance (e.g., Guillot et al., 2009; Hyde et al., 2017; Tomasino et al., 2005; Williams et al., 2012) may be a consequence of other neurophysiological processes and/or activity within upstream brain regions involved in MI performance (i.e., spill over effects).

Further research is therefore needed to explore whether modulation of other cortical and subcortical regions implicated in mental rotation (see this section above) may induce changes in MI performance, and how this influences the activity in the interconnected motor cortices. This study also highlights the need to consider the duration of both TBS application and its related neural effects when attempting to induce cognitive‐behavioral changes in motor areas. Thus, future work should explore whether repeated application of TBS to motor regions across multiple sessions results in enduring changes to PMC activity, and thus alters MI ability. It should also carefully consider the time point participants are completing the behavioral tasks (i.e., HRT and LNRT), to ensure that their administration coincide with the desired inhibitory effects of cTBS. Given the dynamic effects of cTBS, this could be achieved by monitoring corticospinal excitability (via MEPs) beyond the post 5 and 15‐min mark that was adopted in the present study to model the individual response to cTBS. This could also be accomplished by administering the tasks at varying time points following stimulation (e.g., immediately following stimulation).

It is also important to note, that while the current study applied cTBS to the PMC and SMA in accordance with previous literature (Cona et al., 2017; Hamada et al., 2008; Huang et al., 2005; Janssen et al., 2015; Vernet et al., 2013), and recorded MEPs via single‐pulse TMS to indirectly infer whether cTBS had the desired inhibitory effect on PMC excitability, we are unable to confirm whether cTBS had specifically targeted the PMC and SMA or whether adjacent motor cortices were also stimulated in the process. Future research adopting TBS to examine the involvement of motor cortices in MI should consider using MRI scanning and/or neuronavigational systems to better locate and target the relevant motoric regions of interest. Additional experimental investigations may also want to consider measuring MI performance before and after applying cTBS to motor related regions. While the current study used a sham condition as an indicator of baseline performance, administering behavioral tasks (i.e., HRT) pre‐ and post‐cTBS for each condition/stimulation target may provide further insight into whether cTBS alters MI performance, and by extension, the involvement of motoric regions in MI ability.

## 5 CONCLUSION

The present study showed that cTBS had inhibitory effects on cortical excitability in adults with typical motor ability but induced no changes in HRT and LNRT performance after application to the PMC or SMA across both groups (i.e., typical motor ability and DCD). While these findings replicate earlier studies that showed the PMC may not be involved in MI (e.g., Cona et al., 2017; Dechent et al., 2004), this study extends our knowledge of the relationship between motor regions and imagery ability. Indeed, by implementing a randomized, double blind, sham‐controlled, cross over, offline cTBS protocol, and using MEPs to gauge the effects of cTBS on cortical excitability, this work suggests that the PMC (and SMA) may not be causally implicated in MI. It further highlights the need to consider multi‐session repetitive stimulation protocols and their consequential neural effects when attempting to induce reliable behavioral changes.

## CONFLICT OF INTEREST

None.

## AUTHOR CONTRIBUTIONS

**Pamela Barhoun:** Conceptualization; data curation; formal analysis; investigation; methodology; project administration; software; visualization; writing – original draft; writing – review and editing. **Ian Fuelscher:** Conceptualization; investigation; methodology; supervision; validation; writing – review and editing. **Michael Do:** Investigation; methodology; resources; validation; visualization; writing – review and editing. **Jason L. He:** Investigation; methodology; software; writing – review and editing. **Andris Cerins:** Investigation; writing – review and editing. **Soukayna Bekkali:** Investigation; writing – review and editing. **George J. Youssef:** Formal analysis; validation; visualization; writing – review and editing. **Daniel Corp:** Investigation; methodology; writing – review and editing. **Brendan P. Major:** Investigation; writing – review and editing. **Dwayne Meaney:** Investigation; writing – review and editing. **Peter G. Enticott:** Conceptualization; writing – review and editing. **Christian Hyde:** Conceptualization; investigation; methodology; project administration; resources; software; supervision; validation; writing – original draft; writing – review and editing.
