# Supplementary material for: The role of the primary motor cortex in motor imagery: A theta burst stimulation study
Source: Psychophysiology. 2022 May 3;59(10):e14077. doi: 10.1111/psyp.14077 (PMC9540768; doi:10.1111/psyp.14077)
Supplement: Supplementary file 1 [file PSYP-59-e14077-s001.docx]

Supplementary Materials

**A**

**Age and Sex Comparison**

Analysis failed to show a significant difference in age between groups, *t* (14.89) = 0.11, *p* = .911. No significant difference was detected in the proportion of males and females in each group, $\chi$^2^ (1, 35) = 2.94, *p* = .086. While the latter appears to approach significance and likely a factor of small sample size, we were unable to covary for sex given that there was only one male participant with DCD in the current study.

**B**

| **Table 1**  *Linear Mixed Model of Pulse Type for Active PMC Stimulation* | | | | | |
| --- | --- | --- | --- | --- | --- |
|  | **Model 1: PMC MEP Time Point x Pulse Type** | | | | |
|  |  | 95% Confidence Interval | |  |  |
| *Predictors* | *Estimates* (*B*) | *Lower* | *Upper* | *t* | *p* |
| Intercept | 1.41  (0.17) | 1.08 | 1.74 | 8.32 | **< .001** |
| Post cTBS 5 min | -0.37  (0.1) | -0.56 | -0.18 | -3.76 | **< .001** |
| Post cTBS 15 min | 0.05  (0.1) | -0.15 | 0.24 | 0.46 | .647 |
| Monophasic Pulse | -0.06  (0.23) | -0.51 | 0.39 | -0.26 | .791 |
| Post cTBS 5 min x Monophasic Pulse | 0.2  (0.13) | -0.06 | 0.46 | 1.51 | .131 |
| Post cTBS 15 min x Monophasic Pulse | -0.22  (0.13) | -0.48 | 0.04 | -1.67 | .095 |
| **Random Effects** | | | | | |
| σ^2^ | 1.26 | | | | |
| τ_00_ _subject_ | 0.31 | | | | |
| ICC | 0.2 | | | | |
| N _subject_ | 29 | | | | |
| Observations | 1740 | | | | |
| Marginal R^2^ / Conditional R^2^ | 0.013 / 0.208 | | | | |
| *Note.* Standard error presented in brackets; Comparison level for ‘MEP time point’ = pre cTBS; Comparison level for ‘pulse type’ = Biphasic pulse; cTBS = Continuous theta burst stimulation; PMC = Primary motor cortex; MEP = Motor evoked potential. | | | | | |

**C**

**Blinding Procedure**

Participants attended three separate sessions, the order of which was randomised using an automated algorithm. Both the participant and researcher administering the stimulation were blinded to each condition (i.e., active or sham). The order of sessions for each participant was documented in a spreadsheet and was concealed from the leading researcher administering the cTBS throughout the duration of the study. Prior to each session, an assisting research member (i.e., not administering the cTBS) would access the spreadsheet to determine the appropriate condition and site corresponding to that particular session and participant. The researcher administering the cTBS would only be notified about the site of the stimulation (i.e., PMC or SMA), but not the condition (i.e., active or sham). The two figure-of-eight air film cooled coils that delivered active or sham stimulation were placed outside the testing room before commencing each session. Thus, prior to cTBS application, the assisting researcher would leave the testing room to acquire the relevant coil and activate the cTBS machine. The researcher administering the cTBS would then be given the appropriate coil and would apply the stimulation to the relevant site (i.e., PMC or SMA). Both coils were identical in appearance, ensuring the researcher administering the cTBS would be unable to determine the type of stimulation being delivered (i.e., active or sham). The assisting researchers, however, were able to determine the type of stimulation that each coil produced by using a method that was obscured from the researcher administering the cTBS throughout the study.

**D**

**Figure 1**

*Responses Provided Following Stimulation*


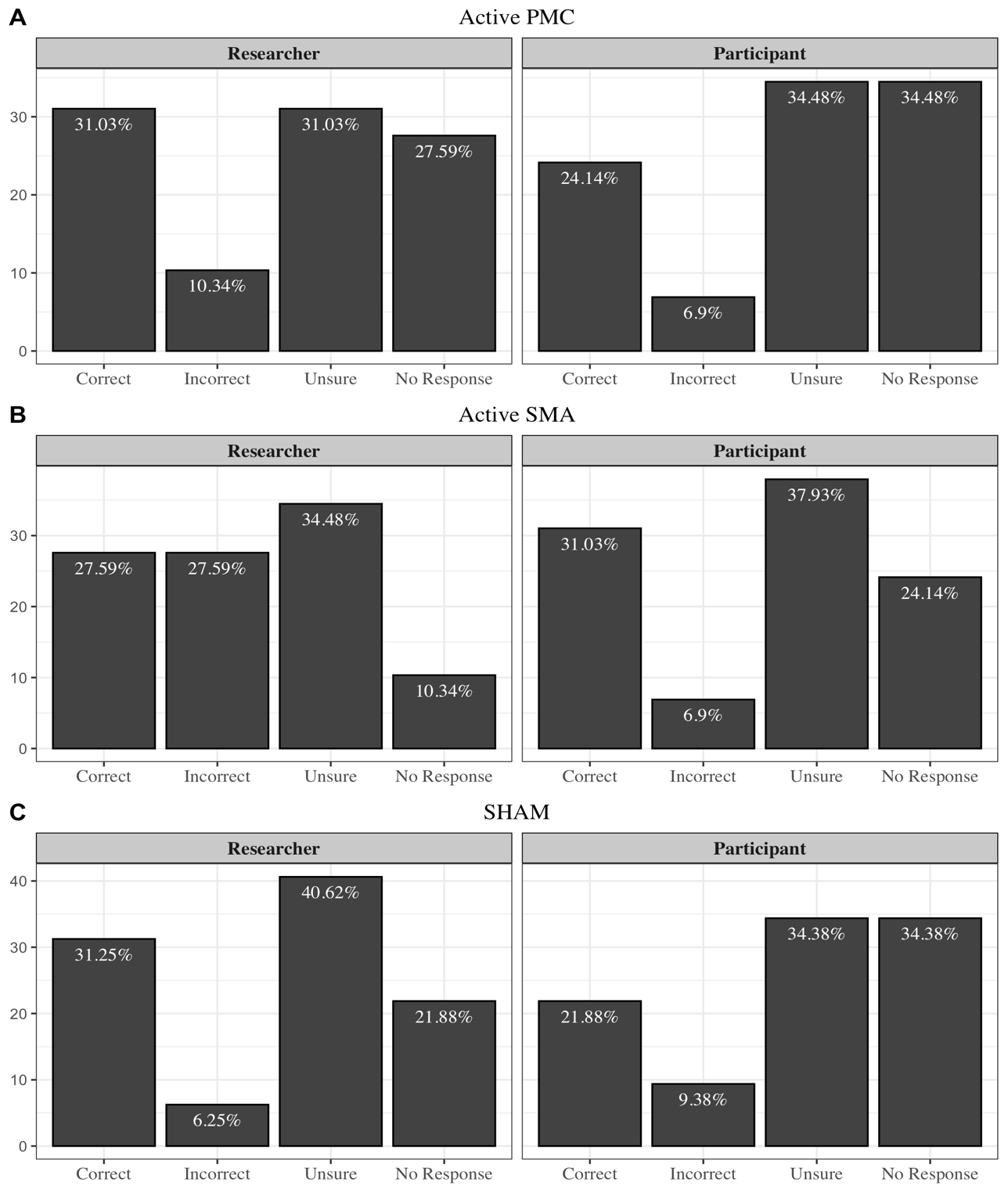


*Note.* PMC = Primary motor cortex; SMA = Supplementary motor area; Correct = Percentage of individuals that listed ‘active stimulation’ following an active PMC or SMA session, or listed ‘sham stimulation’ following a sham session; Incorrect = Percentage of individuals that listed ‘sham stimulation’ following an active PMC or SMA session, or listed ‘active stimulation’ following a sham session; Unsure = Percentage of individuals that listed ‘unsure’ following a session; No Response = Percentage of responses that were not provided following an active PMC or SMA, or sham session.

**E**

**Observed Power Levels**

We calculated the observed power levels for our models reported in Table 5, 6, 7, 11 and 12 in supplementary material J and M using the R package SIMR (Green & MacLeod, 2016). Cohen’s *d*_z_ effect size values were calculated using the MOTE Effect Size Calculator from the DOOM lab (Buchanan et al., 2017).

For the main effect of MEP time point (Model 1 Table 5, 6 and 7), observed power levels were 93.50% (DV = MEPs Sham), 97.00% (DV = MEPs ‘active PMC’) and 84.00% (DV = MEPs ‘active SMA’). Based on the Cohen’s *d*_z_ values, MEP time point showed a small and medium effect on MEPs for the sham (Post cTBS 5 min = -.12; post cTBS 15 min = .50), ‘active PMC’ (Post cTBS 5 min = -.69; post cTBS 15 min = -.21), and ‘active SMA’ conditions (Post cTBS 5 min = -.37; post cTBS 15 min = .15). For the MEP time point x group interaction (Model 2 Table 5, 6 and 7), observed power levels were 48.50% (DV = MEPs Sham), 99.50% (DV = MEPs ‘active PMC’) and 33.00% (DV = MEPs ‘active SMA’). Based on Cohen’s *d*_z_ values, the MEP time point x group interactions demonstrated a small effect for the sham (Post cTBS 5 min x group = .38; post cTBS 15 min x group = .18) and ‘active SMA’ conditions (Post cTBS 5 min x group = -.26; post cTBS 15 min x group = -.32), and a small to medium effect for the ‘active PMC’ condition (Post cTBS 5 min x group = .63; post cTBS 15 min x group = -.26).

For the main effect of condition (Model 1 Table 11 and 12), observed power levels were 16% (DV = HRT efficiency) and 9.50% (DV = LNRT efficiency). Based on the Cohen’s *d*_z_ values, condition showed a small effect on HRT (‘active PMC’ = .10; ‘active SMA’ = .21) and LNRT efficiency (‘active PMC’ = .11; ‘active SMA’ = .07). For the condition x group interaction (Model 2 Table 11 and 12), observed power levels were 51% (DV = HRT efficiency) and 41.00% (DV = LNRT efficiency). Based on Cohen’s *d*_z_ values, the condition x group interaction displayed a small and medium effect on HRT (‘active PMC’ x group = -.06; ‘active SMA’ x group = .37) and LNRT efficiency (‘active PMC’ = .34; ‘active SMA’ = .18).

Based on the observed power, we were largely able to reliably detect small-medium effects across our analyses but were underpowered to detect small effects. It is important to note, however, that these observed power calculations are based on the detected effects in the current data, and thus are influenced by the observed significance levels of each test (Hoenig & Heisey, 2001). Indeed, work has shown that non-significant effects (i.e., those with non-significant *p* values > .05) will correspond to low experimental power (Hoenig & Heisey, 2001). Moreover, the use of the observed effects in estimating power assumes that the current sample has zero sampling error; an assumption that is overly optimistic and thus it is unreasonable to assume that the effect sizes obtained are the exact effect sizes that would occur in the population (Hoenig & Heisey, 2001; Perugini et al., 2018). When coupled together, given that the present effects (i.e., the effect of condition on HRT and LNRT performance) were found to be non-significant, and that post-hoc power computations may provide inaccurate evaluations (Hoenig & Heisey, 2001; Perugini et al., 2018) since these are based on the observed effect sizes (instead of more reasonable assumptions for the size of an effect in the population that is of a size worth examining), we urge caution in interpreting these observed power calculations.

**F**

**Efficiency Metric Assumptions**

The efficiency metric is only appropriate for ‘choice reaction time’ tasks when (a) a positive association between RT and angular disparity, and negative association between accuracy and angular disparity is displayed, and (b) if the independent variables explain more variance in efficiency than RT (Bruyer & Brysbaert, 2011; Townsend & Ashby, 1978, 1983).

In response to criterion ‘a’, trend analyses revealed a significant positive linear trend between angular rotation and RT for both groups on the HRT for the sham condition. That is, mean RT increased with angle of rotation for the typically developing group, *B* = 329.17, *SE* = 64.54, 95% CI [200.99, 457.36], *t* (92) = 5.10, *p* < .001, and for the DCD group, *B* = 777.59, *SE* = 94.08, 95% CI [590.73, 964.45], *t* (92) = 8.26, *p* < .001. A significant positive linear trend was also observed on the LNRT for the sham condition across both groups, *B* = 373.236, *SE* = 39.51, 95% CI [295.80, 450.67], *t* (112) = 9.45, *p* < .001. Further, trend analyses revealed a significant negative trend between angular rotation and accuracy across both groups on the HRT for the sham condition. That is, mean accuracy decreased with angle of rotation for both the typically developing and DCD groups, *B* = -.07, *SE* = .03, 95% CI [-.11, -.02], *t* (92) = -2.59, *p* = .011. This significant negative trend was also observed on the LNRT for the sham condition for the typically developing group, *B* = -.14, *SE* = .03, 95% CI [-.19, -.09], *t* (112) = -5.38, *p* < .001, and the DCD group, *B* = -.25, *SE* = .04, 95% CI [-.32, -.17], *t* (112) = -6.23, *p* < .001.

In response to criterion ‘b’, linear mixed models for the HRT indicated the fixed effects (condition and condition x group) explained more variance in RT (Marginal *R*^2^ = .109) than in efficiency (Marginal *R*^2^ = .074). Linear mixed models for the LNRT also revealed the fixed effects (condition and condition x group) explained more variance in RT (Marginal *R*^2^ = .064) than in efficiency (Marginal *R*^2^ = .038). While criterion ‘b’ was not sufficiently met, further analyses displayed similar results when using RT compared to efficiency. Indeed, after removing one extreme score from the DCD group for the ‘active SMA’ condition on the HRT, and one extreme score from the DCD group for both the ‘active PMC’ and ‘active SMA’ condition on the LNRT (see Figure 2 below), results showed no significant changes in HRT and LNRT performance following active stimulation to either the PMC or SMA compared to the sham condition when using the RT metric (see Table 2 and 3). Given that no differences were detected between models using either the RT or efficiency metric, we felt confident using efficiency as the primary measure of performance in the current study.

**Figure 2**


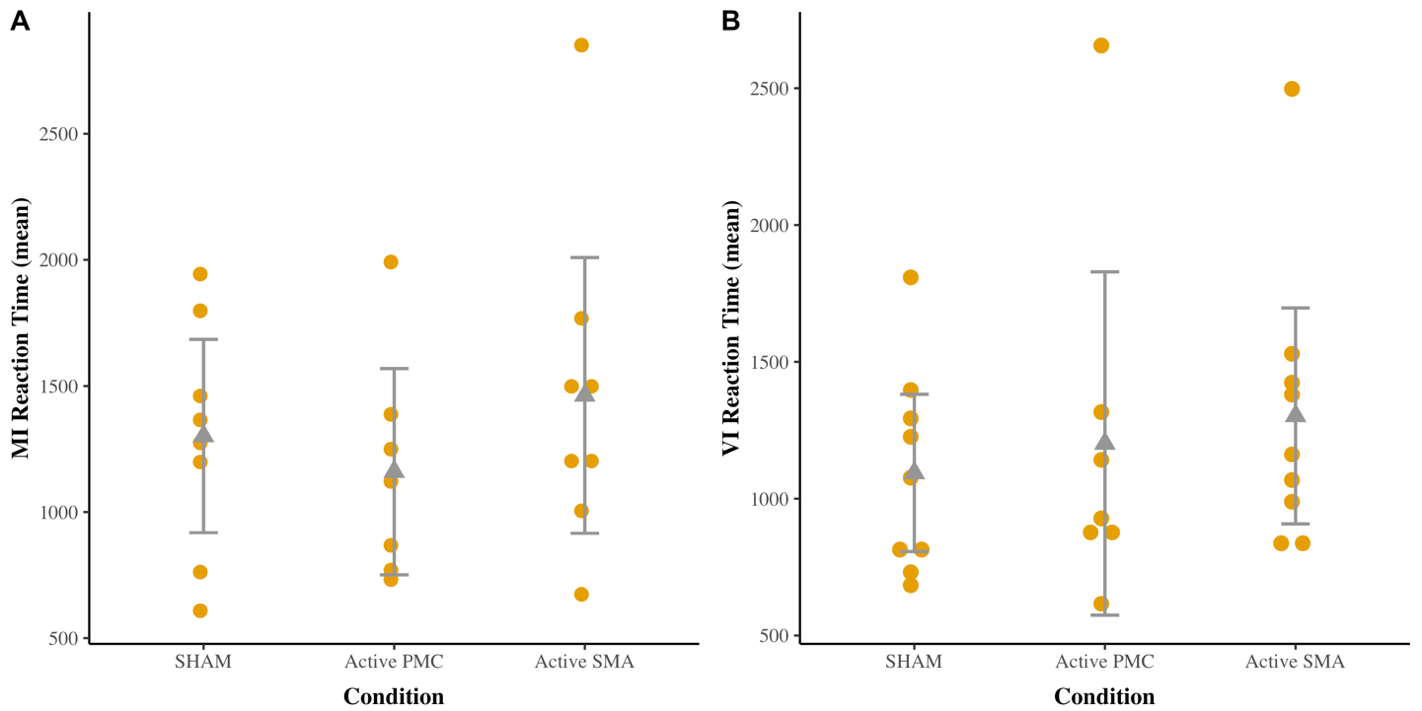
*Individual Data Points for MI and VI Reaction Time for Individuals with DCD Across Conditions*

*Note.* Sample means and lower and upper 95% confidence intervals presented; MI = Motor imagery; VI = Visual imagery; **A**. MI efficiency as indicated by performance on the hand rotation task; **B.** VI efficiency as indicated by performance on the letter number rotation task.

| **Table 2**  *Linear Mixed Models for Motor Imagery Performance (Reaction Time) Across Conditions* | | | | | | | | | | | |
| --- | --- | --- | --- | --- | --- | --- | --- | --- | --- | --- | --- |
|  | Model 1: Condition | | | | |  | Model 2: Condition x Group | | | | |
|  |  | 95% Confidence Interval | |  |  |  |  | 95% Confidence Interval | |  |  |
| *Predictors* | *Estimates* (*B*) | *Lower* | *Upper* | *t* | *p* |  | *Estimates* (*B*) | *Lower* | *Upper* | *t* | *p* |
| Intercept | 1063.54 (73.49) | 919.50 | 1207.58 | 14.47 | **< .001** |  | 940.87 (83.46) | 777.28 | 1104.45 | 11.27 | **< .001** |
| Active PMC | 42.41 (51.25) | -58.04 | 142.87 | 0.83 | .408 |  | 101.72 (61.78) | -19.35 | 222.80 | 1.65 | .100 |
| Active SMA | 59.47 (51.98) | -42.42 | 161.36 | 1.14 | .253 |  | 64.55 (61.78) | -56.52 | 185.63 | 1.04 | .296 |
| Active PMC x Group (DCD) |  |  |  |  |  |  | -188.17 (109.81) | -403.39 | 27.05 | -1.71 | .087 |
|  |  |  |  |  |  |  |  |  |  |  |  |
| Active SMA x Group (DCD) |  |  |  |  |  |  | -12.41 (113.32) | -234.52 | 209.70 | -0.11 | .913 |
|  |  |  |  |  |  |  |  |  |  |  |  |
| **Random Effects** | | | | | | | | | | | |
| σ^2^ | 147056.45 | | | | |  | 146368.64 | | | | |
| τ_00_ | 115529.59 _subject_ | | | | |  | 97061.00 _subject_ | | | | |
| ICC | 0.44 | | | | |  | 0.40 | | | | |
| N | 28 _subject_ | | | | |  | 28 _subject_ | | | | |
| Observations | 355 | | | | |  | 355 | | | | |
| Marginal R^2^ / Conditional R^2^ | 0.002 / 0.441 | | | | |  | 0.085 / 0.450 | | | | |
| *Note.* Standard error presented in brackets; Comparison level for ‘condition’ = Sham; Comparison level for ‘group’ = Control; PMC = Primary motor cortex; SMA = Supplementary motor area; DCD = Developmental Coordination Disorder. | | | | | | | | | | | |

| **Table 3**  *Linear Mixed Models for Visual Imagery Performance (Reaction Time) Across Conditions* | | | | | | | | | | | |
| --- | --- | --- | --- | --- | --- | --- | --- | --- | --- | --- | --- |
|  | Model 1: Condition | | | | |  | Model 2: Condition x Group | | | | |
|  |  | 95% Confidence Interval | |  |  |  |  | 95% Confidence Interval | |  |  |
| *Predictors* | *Estimates* (*B*) | *Lower* | *Upper* | *t* | *p* |  | *Estimates* (*B*) | *Lower* | *Upper* | *t* | *p* |
| Intercept | 1046.82 (59.97) | 929.29 | 1164.36 | 17.46 | **< .001** |  | 1012.86 (71.07) | 873.57 | 1152.15 | 14.25 | **< .001** |
| Active PMC | -47.26 (37.14) | -120.05 | 25.52 | -1.27 | .203 |  | -70.01 (43.02) | -154.32 | 14.31 | -1.63 | .104 |
| Active SMA | 9.73 (36.52) | -61.84 | 81.31 | 0.27 | .790 |  | -32.18 (43.02) | -116.49 | 52.13 | -0.75 | .454 |
| Active PMC x Group (DCD) |  |  |  |  |  |  | 85.52 (84.80) | -80.68 | 251.73 | 1.01 | 0.313 |
|  |  |  |  |  |  |  |  |  |  |  |  |
| Active SMA x Group (DCD) |  |  |  |  |  |  | 148.10 (80.87) | -10.40 | 306.59 | 1.83 | .067 |
|  |  |  |  |  |  |  |  |  |  |  |  |
| **Random Effects** | | | | | | | | | | | |
| σ^2^ | 85005.25 | | | | |  | 84599.66 | | | | |
| τ_00_ | 98545.59 _subject_ | | | | |  | 96289.05 _subject_ | | | | |
| ICC | 0.54 | | | | |  | 0.53 | | | | |
| N | 33 _subject_ | | | | |  | 33 _subject_ | | | | |
| Observations | 410 | | | | |  | 410 | | | | |
| Marginal R^2^ / Conditional R^2^ | 0.003 / 0.538 | | | | |  | 0.044 / 0.553 | | | | |
| *Note.* Standard error presented in brackets; Comparison level for ‘condition’ = Sham; Comparison level for ‘group’ = Control; PMC = Primary motor cortex; SMA = Supplementary motor area; DCD = Developmental Coordination Disorder. | | | | | | | | | | | |

**G**

| **Table 4**  *Linear Mixed Models for Motor Imagery Performance (Efficiency) Across Conditions (Individuals with no SHAM condition removed)* | | | | | | | | | | | | | |
| --- | --- | --- | --- | --- | --- | --- | --- | --- | --- | --- | --- | --- | --- |
|  | Model 1: Condition | | | | | |  | Model 2: Condition x Group | | | | | |
|  |  | | 95% Confidence Interval | |  |  |  |  | | 95% Confidence Interval | |  |  |
| *Predictors* | *Estimates* (*B*) | | *Lower* | *Upper* | *t* | *p* |  | *Estimates* (*B*) | | *Lower* | *Upper* | *t* | *p* |
| Intercept | 1223.29  (124.80) | | 978.68 | 1467.89 | 9.80 | **< .001** |  | 1120.09  (144.93) | | 836.04 | 1404.15 | 7.73 | **< .001** |
| Active PMC | 42.68  (89.27) | | -132.29 | 217.65 | 0.48 | .633 |  | 59.51  (107.58) | | -151.35 | 270.37 | 0.55 | .580 |
| Active SMA | 89.25  (89.27) | | -85.72 | 264.22 | 1.00 | .317 |  | -24.73  (107.58) | | -235.59 | 186.13 | -0.23 | .818 |
| Active PMC x Group (DCD) |  | |  |  |  |  |  | -58.68  (190.66) | | -432.37 | 315.02 | -0.31 | .758 |
|  |  |  |  |  |  |  |  |  |  |  |  |  |  |
| Active SMA x Group (DCD) |  | |  |  |  |  |  | 352.45  (190.66) | | -21.24 | 726.14 | 1.85 | .065 |
|  |  |  |  |  |  |  |  |  |  |  |  |  |  |
| **Random Effects** | | | | | | | | | | | | | |
| σ^2^ |  | 444401.69 | | | | |  |  | 440411.32 | | | | |
| τ_00_ |  | 300492.57 _subject_ | | | | |  |  | 268987.64 _subject_ | | | | |
| ICC |  | 0.4 | | | | |  |  | 0.38 | | | | |
| N |  | 25 _subject_ | | | | |  |  | 25 _subject_ | | | | |
| Observations |  | 345 | | | | |  |  | 345 | | | | |
| Marginal R^2^ / Conditional R^2^ |  | 0.002 / 0.404 | | | | |  |  | 0.061 / 0.417 | | | | |
| *Note.* Standard error presented in brackets; Comparison level for ‘condition’ = Sham; Comparison level for ‘group’ = Control; PMC = Primary motor cortex; SMA = Supplementary motor area; DCD = Developmental Coordination Disorder. | | | | | | | | | | | | | |

**H**

**Figure 3**

*
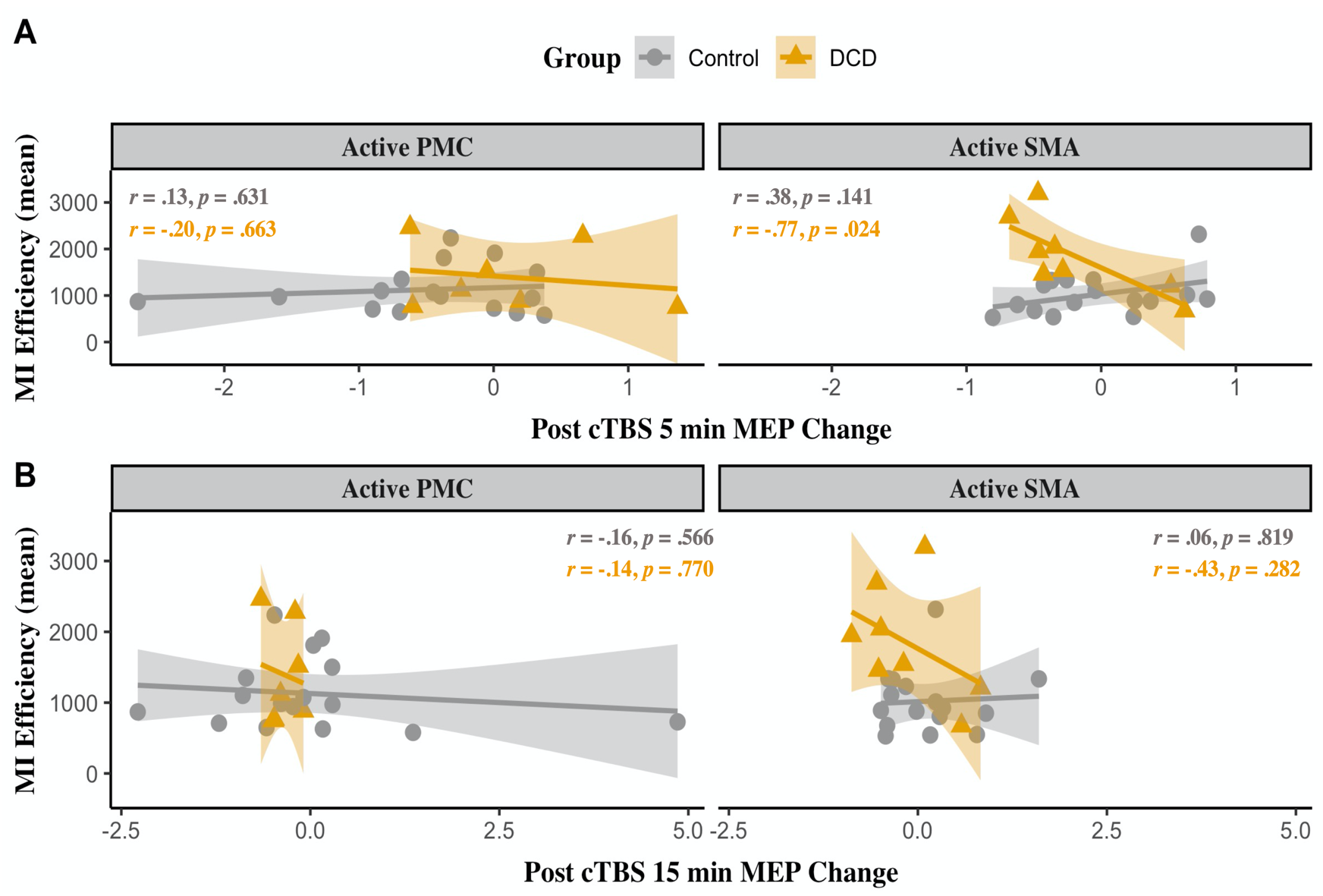
Correlation Between MEP Change and MI Efficiency Across Conditions*

*Note.* MI efficiency as indicated by hand rotation task performance; Bands around the regression lines represent the 95% confidence interval; MI = Motor imagery; PMC = Primary motor cortex; SMA = Supplementary motor area; DCD = Developmental Coordination Disorder; MEP = Motor evoked potential; **A**. MEP change = change scores calculated between MEPs at pre cTBS and post cTBS 5 min; **B.** MEP change = change scores calculated between MEPs at pre cTBS and post cTBS 15 min.

It is important to note that while a significant correlation between post cTBS 5 min MEP change and MI efficiency for ‘active SMA’ has been detected for individuals with DCD, this is likely a result of an extreme data point influencing the slope of the relationship. Specifically, once removing this participant, the correlation between post cTBS 5 min MEP change and MI efficiency for the DCD group is non-significant (*r* = - .64, *p* = .125). Given the limited number of participants with DCD for this analysis (*n* = 8) and the large variability in scores, we urge caution in over interpreting this relationship (i.e., *r* = -.77).

**I**

**Figure 4**


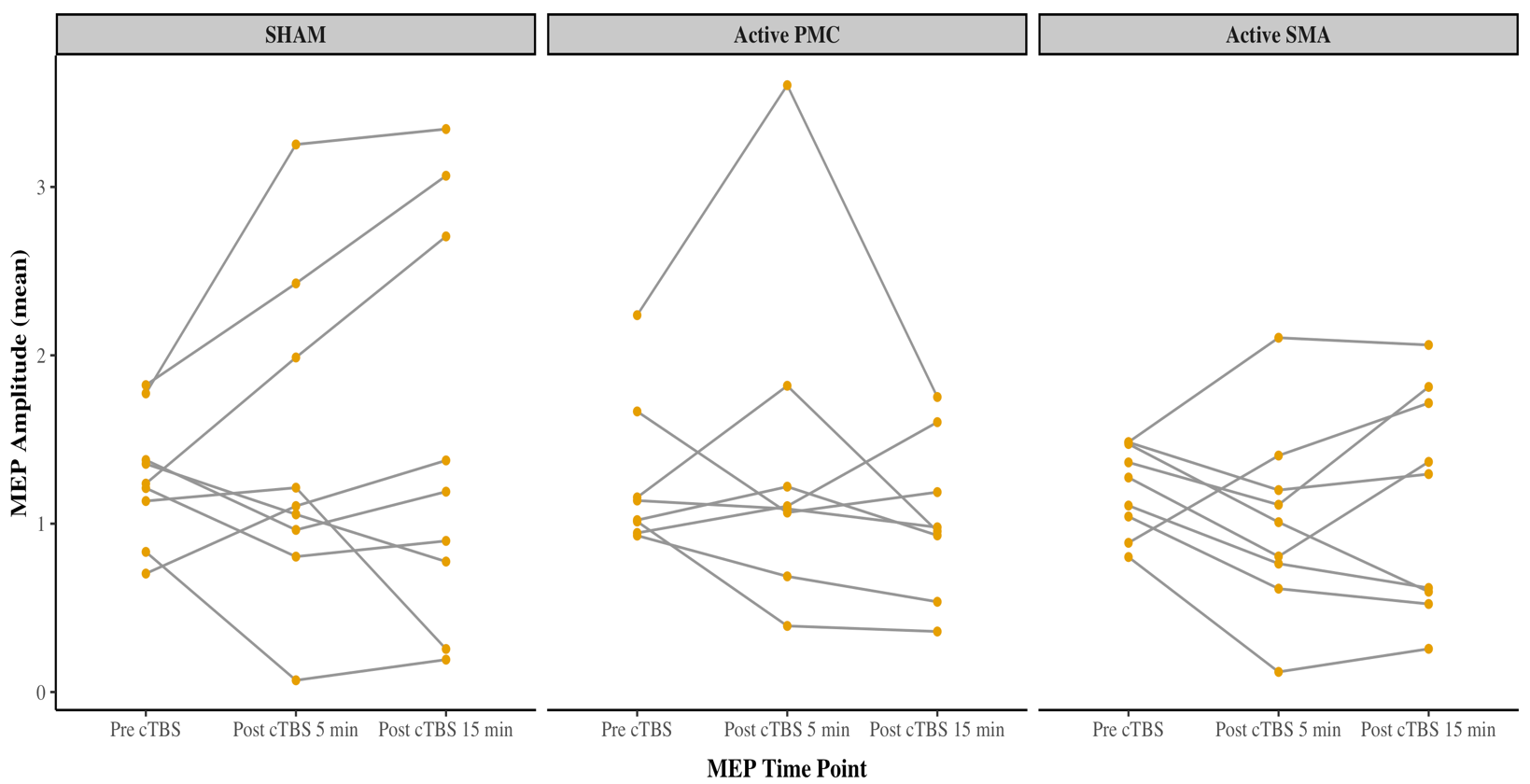
*Individual Data Points for MEP Amplitudes Across MEP Time Point and Condition for Individuals with DCD*

PMC = Primary motor cortex; SMA = Supplementary motor area; DCD = Developmental Coordination Disorder; MEP = Motor evoked potential.

| **Table 5**  *Linear Mixed Models of MEP Time Point for Active PMC Stimulation* | | | | | | | | | | | | |
| --- | --- | --- | --- | --- | --- | --- | --- | --- | --- | --- | --- | --- |
|  | Model 1: Active PMC MEP Time Point | | | | |  | Model 2: Active PMC MEP Time Point x Group | | | | | |
|  |  | 95% Confidence Interval | |  |  |  |  | 95% Confidence Interval | |  |  |  |
| *Predictors* | *Estimates* (*B*) | *Lower* | *Upper* | *t* | *p* |  | *Estimates* (*B*) | *Lower* | *Upper* | *t* | *p* |  |
| Intercept | 1.38  (0.11) | 1.16 | 1.60 | 12.31 | **< .001** |  | 1.42  (0.13) | 1.16 | 1.68 | 10.64 | **< .001** |  |
| Post cTBS 5 min | -0.26  (0.07) | -0.39 | -0.13 | -3.94 | **< .001** |  | -0.40  (0.08) | -0.55 | -0.25 | -5.20 | **< .001** |  |
| Post cTBS 15 min | -0.08  (0.07) | -0.21 | 0.05 | -1.16 | .245 |  | -0.02  (0.08) | -0.17 | 0.13 | -0.26 | .795 |  |
| Group (DCD) |  |  |  |  |  |  | -0.16  (0.25) | -0.65 | 0.34 | -0.61 | .539 |  |
| Post cTBS 5 min x Group (DCD) |  |  |  |  |  |  | 0.51  (0.15) | 0.22 | 0.80 | 3.48 | **.001** |  |
| Post cTBS 15 min x Group (DCD) |  |  |  |  |  |  | -0.21  (0.15) | -0.49 | 0.08 | -1.40 | .161 |  |
| **Random Effects** | | | | | | | | | | | | |
| σ^2^ | 1.26 | | | | |  | 1.25 | | | | | |
| τ_00_ | 0.30 _subject_ | | | | |  | 0.31 _subject_ | | | | | |
| ICC | 0.19 | | | | |  | 0.2 | | | | | |
| N | 29 _subject_ | | | | |  | 29 _subject_ | | | | | |
| Observations | 1740 | | | | |  | 1740 | | | | | |
| Marginal R^2^ / Conditional R^2^ | 0.008 / 0.198 | | | | |  | 0.019 / 0.215 | | | | | |
| *Note.* Standard error presented in brackets; Comparison level for ‘MEP time point’ = pre cTBS; Comparison level for ‘group’ = Control; cTBS = Continuous theta burst stimulation; PMC = Primary motor cortex; DCD = Developmental Coordination Disorder. | | | | | | | | | | | | |

**J**

| **Table 6**  *Linear Mixed Models of MEP Time Point for SHAM Stimulation* | | | | | | | | | | | | |
| --- | --- | --- | --- | --- | --- | --- | --- | --- | --- | --- | --- | --- |
|  | Model 1: SHAM MEP Time Point | | | | |  | Model 2: SHAM MEP Time Point x Group | | | | | |
|  |  | 95% Confidence Interval | |  |  |  |  | 95% Confidence Interval | |  |  |  |
| *Predictors* | *Estimates* (*B*) | *Lower* | *Upper* | *t* | *p* |  | *Estimates* (*B*) | *Lower* | *Upper* | *t* | *p* |  |
| Intercept | 1.22  (0.10) | 1.03 | 1.41 | 12.58 | **< .001** |  | 1.20  (0.11) | 0.98 | 1.42 | 10.50 | **< .001** |  |
| Post cTBS 5 min | -0.04  (0.06) | -0.15 | 0.07 | -0.74 | .457 |  | -0.12  (0.07) | -0.25 | 0.01 | -1.80 | .071 |  |
| Post cTBS 15 min | 0.17  (0.06) | 0.06 | 0.28 | 2.95 | **.003** |  | 0.13  (0.07) | -0.00 | 0.26 | 1.95 | .051 |  |
| Group (DCD) |  |  |  |  |  |  | 0.07  (0.22) | -0.35 | 0.50 | 0.34 | .735 |  |
| Post cTBS 5 min x Group (DCD) |  |  |  |  |  |  | 0.28  (0.13) | 0.03 | 0.53 | 2.21 | **.027** |  |
| Post cTBS 15 min x Group (DCD) |  |  |  |  |  |  | 0.13  (0.13) | 0.12 | 0.38 | 1.03 | .301 |  |
| **Random Effects** | | | | | | | | | | | | |
| σ^2^ | 1.03 | | | | |  | 1.03 | | | | | |
| τ_00_ | 0.25 _subject_ | | | | |  | 0.25 _subject_ | | | | | |
| ICC | 0.19 | | | | |  | 0.19 | | | | | |
| N | 32 _subject_ | | | | |  | 32 _subject_ | | | | | |
| Observations | 1920 | | | | |  | 1920 | | | | | |
| Marginal R^2^ / Conditional R^2^ | 0.006 / 0.199 | | | | |  | 0.015 / 0.206 | | | | | |
| *Note.* Standard error presented in brackets; Comparison level for ‘MEP time point’ = pre cTBS; Comparison level for ‘group’ = Control; cTBS = Continuous theta burst stimulation; DCD = Developmental coordination disorder. | | | | | | | | | | | | |

| **Table 7**  *Linear Mixed Models of MEP Time Point for Active SMA Stimulation* | | | | | | | | | | | | |
| --- | --- | --- | --- | --- | --- | --- | --- | --- | --- | --- | --- | --- |
|  | Model 1: Active SMA MEP Time Point | | | | |  | Model 2: Active SMA MEP Time Point x Group | | | | | |
|  |  | *95% Confidence Interval* | |  |  |  |  | *95% Confidence Interval* | |  |  |  |
| *Predictors* | *Estimates* (*B*) | *Lower* | *Upper* | *t* | *p* |  | *Estimates* (*B*) | *Lower* | *Upper* | *t* | *p* |  |
| Intercept | 1.11  (0.08) | 0.96 | 1.27 | 14.37 | **< .001** |  | 1.07  (0.09) | 0.88 | 1.26 | 11.29 | **< .001** |  |
| Post cTBS 5 min | -0.10  (0.05) | -0.20 | -0.01 | -2.14 | **.032** |  | -0.06  (0.06) | -0.17 | 0.05 | -1.02 | .309 |  |
| Post cTBS 15 min | 0.04  (0.05) | -0.05 | 0.13 | 0.87 | .385 |  | 0.09  (0.06) | -0.02 | 0.21 | 1.63 | .103 |  |
| Group (DCD) |  |  | |  |  |  | 0.14  (0.17) | -0.19 | 0.48 | 0.84 | .399 |  |
| Post cTBS 5 min x Group (DCD) |  |  | |  |  |  | -0.14  (0.10) | -0.34 | 0.06 | -1.36 | .173 |  |
| Post cTBS 15 min x Group (DCD) |  |  | |  |  |  | -0.17  (0.10) | -0.37 | 0.03 | -1.64 | .102 |  |
| **Random Effects** | | | | | | | | | | | | |
| σ^2^ | 0.66 | | | | |  | 0.66 | | | | | |
| τ_00_ | 0.14 _subject_ | | | | |  | 0.15 _subject_ | | | | | |
| ICC | 0.18 | | | | |  | 0.18 | | | | | |
| N | 29 _subject_ | | | | |  | 29 _subject_ | | | | | |
| Observations | 1740 | | | | |  | 1740 | | | | | |
| Marginal R^2^ / Conditional R^2^ | 0.005 / 0.181 | | | | |  | 0.006 / 0.188 | | | | | |
| *Note.* Standard error presented in brackets; Comparison level for ‘MEP time point’ = pre cTBS; Comparison level for ‘group’ = Control; cTBS = Continuous theta burst stimulation; SMA = Supplementary motor area; DCD = Developmental Coordination Disorder. | | | | | | | | | | | | |

**K**

| **Table 8**  *Descriptive Statistics for Reaction Time and Accuracy Metrics for Motor and Visual Imagery Performance Across Conditions* | | | | | | | |
| --- | --- | --- | --- | --- | --- | --- | --- |
|  | HRT | | |  | LNRT | | |
| *Condition* | *Sample Size* | *RT (ms)* | *Accuracy* |  | *Sample Size* | *RT (ms)* | *Accuracy* |
| **SHAM**  Control  DCD | 17  8 | 914.92  (44.66)  1301.37  (100.43) | 0.90  (0.02)  0.93  (0.03) |  | 21  9 | 1033.57  (41.28)  1093.94  (63.55) | 0.90  (0.01)  0.84  (0.03) |
| **Active PMC**  Control  DCD | 16  7 | 994.11  (46.33)  1159.90  (92.49) | 0.93  (0.01)  0.91  (0.03) |  | 19  7 | 908.28  (36.10)  1201.68  (129.56) | 0.90  (0.02)  0.87  (0.03) |
| **Active SMA**  Control  DCD | 16  8 | 958.29  (52.26)  1462.43  (119.80) | 0.95  (0.01)  0.87  (0.03) |  | 19  9 | 974.77  (41.73)  1302.65  (88.50) | 0.93  (0.01)  0.88  (0.03) |
| *Note.* Standard error presented in brackets; DCD = Developmental Coordination Disorder; HRT = Hand rotation task (measure of motor imagery); LNRT = Letter number rotation task (measure of visual imagery); PMC = Primary motor cortex; SMA = Supplementary motor area; RT = Reaction time. | | | | | | | |

**Figure 5**

*MI and VI Performance for Controls and Individuals with DCD Across Conditions for Reaction Time*


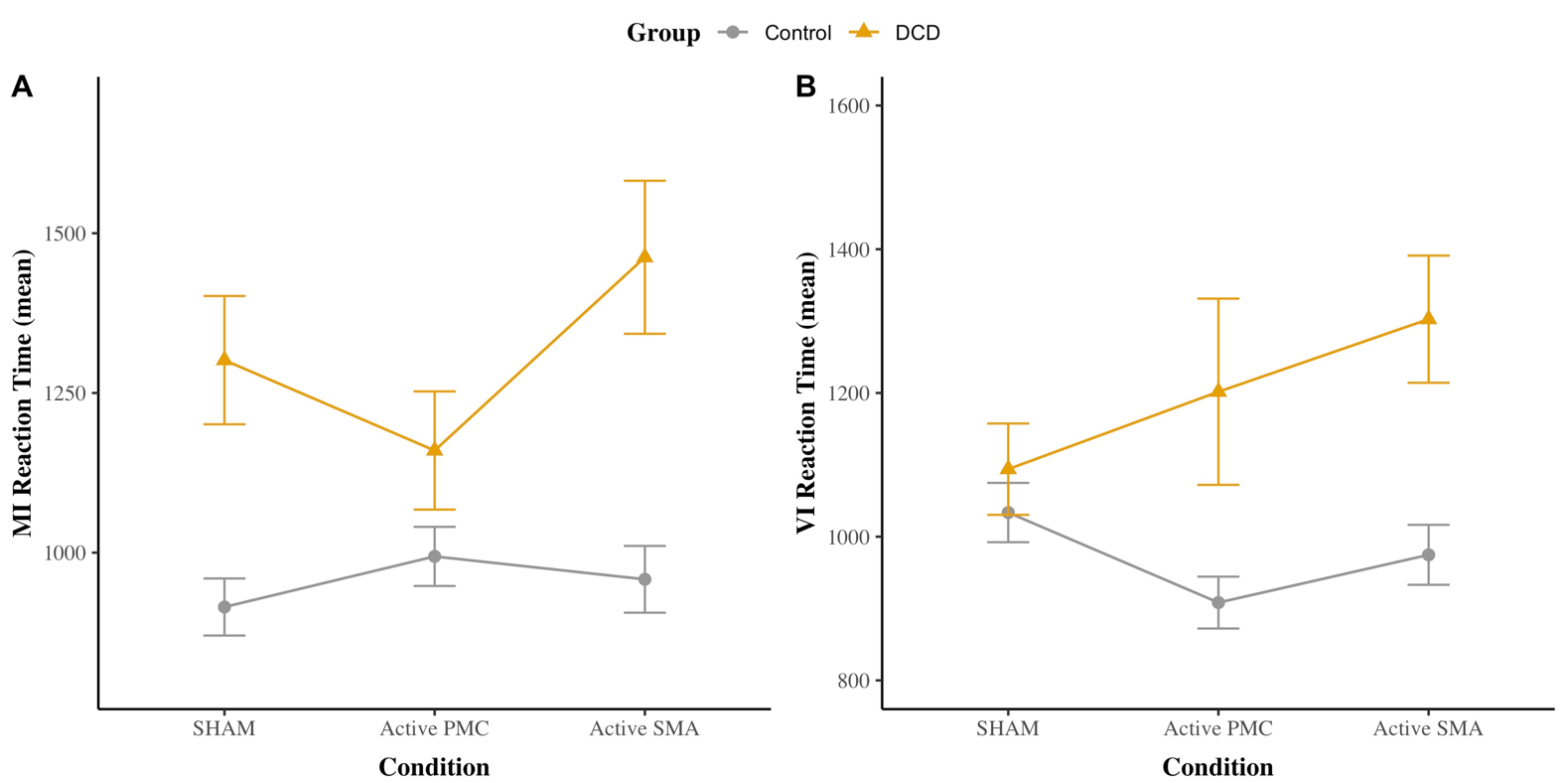


*Note.* Standard error presented; DCD = Developmental Coordination Disorder; MI = Motor imagery; VI = Visual imagery; **A**. MI reaction time as indicated by performance on the hand rotation task; **B.** VI reaction time as indicated by letter number rotation task performance.

**Figure 6**


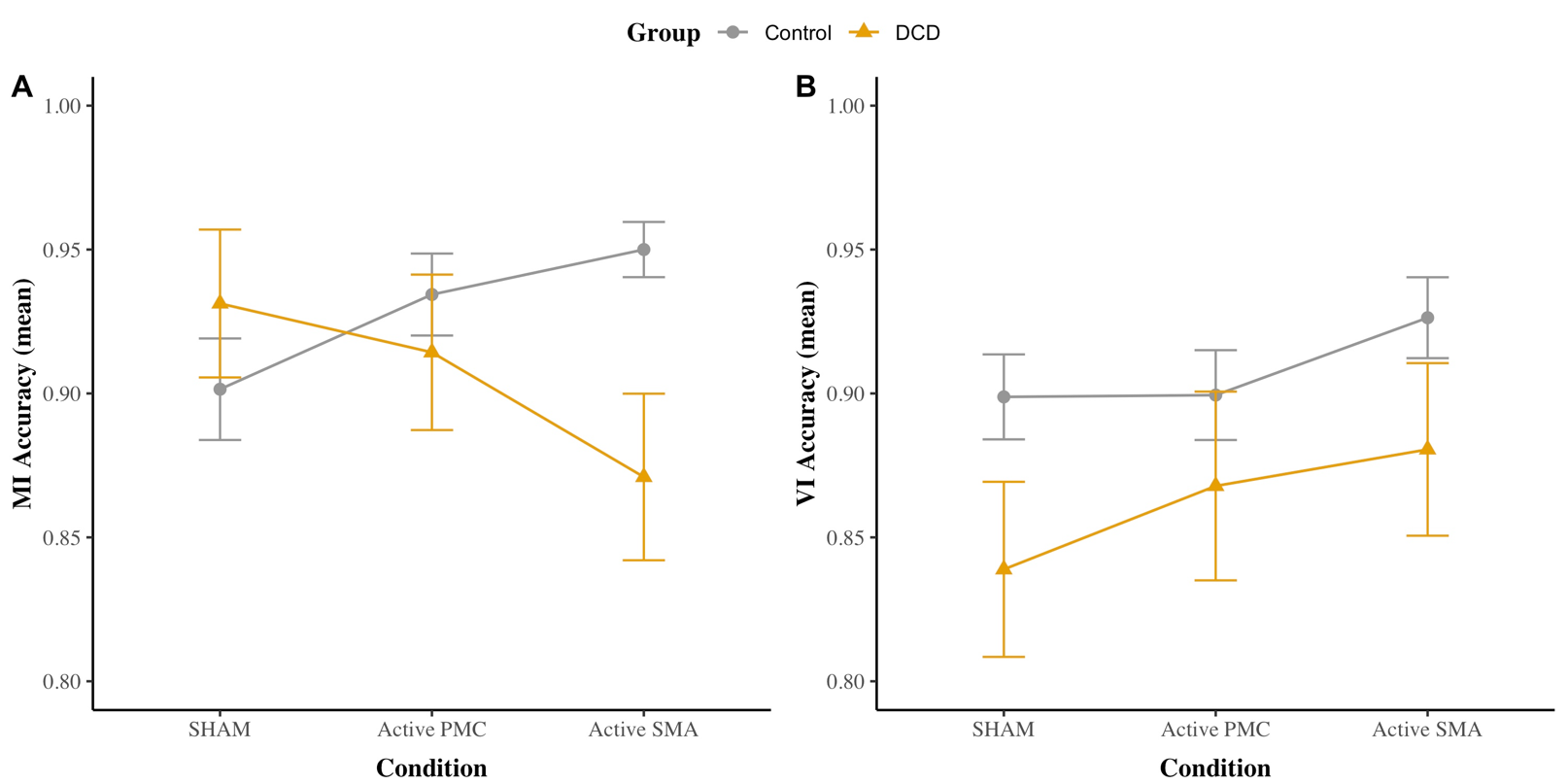
*MI and VI Performance for Controls and Individuals with DCD Across Conditions for Accuracy*

*Note.* Standard error presented; DCD = Developmental Coordination Disorder; MI = Motor imagery; VI = Visual imagery; **A**. MI accuracy as indicated by performance on the hand rotation task; **B.** VI accuracy as indicated by letter number rotation task performance.

**L**

**Order Effects**

As noted in the manuscript, the initial randomisation of the sessions/conditions (‘active PMC’, ‘active SMA’ and sham PMC/SMA) were counterbalanced to be equally distributed across subjects. Likewise, the initial randomisation of the tasks (HRT and LNRT) completed across the sessions were also counterbalanced to be equally distributed across sessions and subjects. However, as some participants were unable to complete all three sessions and due to unforeseeable drop out, not all possible condition or task orders across sessions were completed equally. To examine the possible order effects of completing the tasks across sessions/conditions, separate linear mixed models using restricted maximum likelihood estimation (REML) were conducted for both the HRT and LNRT, with performance efficiency as the dependent variable and with session (session 1 vs. session 2 and session 3) and session x group (control vs DCD) as fixed effects. Both models contained a random intercept to account for clustering of efficiency scores in each session within individuals.

The linear mixed models for HRT performance revealed a statistically significant main effect for session, *F* (2, 337.45) = 4.44, *p* = .013. Specifically, a significant reduction in HRT efficiency in session 3 compared to session 1 across both groups was detected, *B* = -272.45, *SE* = 91.53, 95% CI [-451.85, -93.05], *t* (342.42) = -2.98, *p* = .003, *d*_z_ = -0.56, 95% CI*_d_*_z_ [-0.96, -0.16]. No change was observed in session 2 and no interactions between session and group were observed (see table 9 below). These findings indicate that HRT performance may have improved in session 3 as a result of possible practice effects. Although the current sample is above average for this type of work, it is still modest in size and therefore unable to meaningfully covary for learning effects in session 3. Nevertheless, this supports the possible null effects of the current study discussed in the main manuscript, indicating that 1) the PMC (and SMA) may not be directly involved with MI ability and/or 2) the current cTBS protocol is not optimised to alter behavioural performance during a single session design given that, even if cTBS might potentially impact HRT performance, the effect of downregulating the motor cortices on HRT performance would need to exceed the practice effects observed across multiple sessions for such effects to be detected.

The linear mixed models for LNRT performance revealed no statistically significant main effect for session, (*F* (2, 405.02) = 0.08, *p* = .920). Specifically, no statistically significant effect was detected for session 2 (*B* = -81.53, *SE* = 228.64, 95% CI [-529.66, 366.59], *t* (411.48) = -0.36, *p* = .721, *d*_z_ = -0.06, 95% CI*_d_*_z_ [-0.40, 0.28]) or session 3 (*B* = 2.99, *SE* = 231.77, 95% CI [-451.26, 457.24], *t* (412.42) = 0.01, *p* = .990, *d*_z_ = 0.00, 95% CI*_d_*_z_ [-0.34, 0.34]). This indicated that across all individuals with typical motor ability and DCD, no significant changes in LNRT efficiency were detected in session 2 and session 3 when compared to session 1 (see table 10 below). While the overall test for a significant interaction between session and group was not statistically significant for LNRT performance, *F* (2, 402.53) = 2.03, *p* = .133, it appeared to approach significance for session 3 x group in the model, *B* = 1021.98, *SE* = 509.40, 95% CI [23.56, 2020.39], *t* (411.30) = 2.01, *p* = .045, *d*_z_ = 0.35, 95% CI*_d_*_z_ [0.00 0.70] . Further inspection of the individual data points revealed that an extreme score for a participant with DCD is likely contributing to this effect in the model (please see figure 7 below). Indeed, once removing session 3 for this participant from the model, no interaction between session 3 and group is observed, *B* = 78.49, *SE* = 291.85, 95% CI [-493.53, 650.51], *t* (405.23) = 0.27, *p* = .788, *d*_z_ = 0.05, 95% CI*_d_*_z_ [-0.29, 0.39]).

| **Table 9**  *Linear Mixed Models of Motor Imagery Performance Across Sessions* | | | | | | | | | | | | | |
| --- | --- | --- | --- | --- | --- | --- | --- | --- | --- | --- | --- | --- | --- |
|  | Model 1: Session | | | | | | |  | Model 2: Session x Group | | | | |
|  |  | | | *95% Confidence Interval* | |  |  |  |  | *95% Confidence Interval* | |  |  |
| *Predictors* | *Estimates* (B) | | | *Lower* | *Upper* | *t* | *p* |  | *Estimates* (B) | *Lower* | *Upper* | *t* | *p* |
| Intercept | 1432.73  (117.88) | | | 1201.68 | 1663.78 | 12.15 | **< .001** |  | 1307.1  (134.78) | 1042.93 | 1571.26 | 9.70 | **< .001** |
|  |  |  |  |  |  |  |  |  |  |  |  |  |  |
| Session 2 | -122.69  (91.53) | | | -302.10 | 56.71 | -1.34 | .180 |  | -192.94  (110.88) | -410.26 | 24.38 | -1.74 | .082 |
|  |  |  |  |  |  |  |  |  |  |  |  |  |  |
| Session 3 | -272.45  (91.53) | | | -451.85 | -93.05 | -2.98 | **.003** |  | -314.21  (110.88) | -531.53 | -96.89 | -2.83 | **.005** |
|  |  |  |  |  |  |  |  |  |  |  |  |  |  |
| Group (DCD) |  | | |  |  |  |  |  | 390.86  (237.73) | -75.08 | 856.79 | 1.64 | .100 |
|  |  |  |  |  |  |  |  |  |  |  |  |  |  |
| Session 2 x Group (DCD) |  | | |  |  |  |  |  | 206.91  (196.46) | -178.14 | 591.97 | 1.05 | .292 |
|  |  |  |  |  |  |  |  |  |  |  |  |  |  |
| Session 3 x Group (DCD) |  | | |  |  |  |  |  | 117.38  (196.46) | -267.67 | 502.43 | 0.60 | .550 |
|  |  |  |  |  |  |  |  |  |  |  |  |  |  |
| **Random Effects** | | | | | | | | | | | | | |
| σ^2^ |  | | 473146.23 | | | | |  | 474790.8 | | | | |
| τ_00_ |  | | 294472.58 _subject_ | | | | |  | 250180.10 _subject_ | | | | |
| ICC |  | | 0.38 | | | | |  | 0.35 | | | | |
| N |  | | 28 _subject_ | | | | |  | 28 _subject_ | | | | |
| Observations |  | | 360 | | | | |  | 360 | | | | |
| Marginal R^2^ / Conditional R^2^ | |  | 0.016 / 0.394 | | | | |  | 0.085 / 0.401 | | | | |
| *Note.* Standard error presented in brackets; Comparison level for ‘Session’ = Session 1; Comparison level for ‘group’ = Control; DCD = Developmental Coordination Disorder. | | | | | | | | | | | | | |

| **Table 10**  *Linear Mixed Models of Visual Imagery Performance Across Sessions* | | | | | | | | | | | | | |
| --- | --- | --- | --- | --- | --- | --- | --- | --- | --- | --- | --- | --- | --- |
|  | Model 1: Session | | | | | | |  | Model 2: Session x Group | | | | |
|  |  | | | *95% Confidence Interval* | |  |  |  |  | *95% Confidence Interval* | |  |  |
| *Predictors* | *Estimates* (B) | | | *Lower* | *Upper* | *t* | *p* |  | *Estimates* (B) | *Lower* | *Upper* | *t* | *p* |
| Intercept | 1376.79 (193.20) | | | 998.13 | 1755.46 | 7.13 | **< .001** |  | 1280.79 (222.92) | 843.87 | 1717.71 | 5.75 | **< .001** |
|  |  |  |  |  |  |  |  |  |  |  |  |  |  |
| Session 2 | -81.53 (228.64) | | | -529.66 | 366.59 | -0.36 | .721 |  | -244.64 (272.98) | -779.66 | 290.39 | -0.90 | .370 |
|  |  |  |  |  |  |  |  |  |  |  |  |  |  |
| Session 3 | 2.99 (231.77) | | | -451.26 | 457.24 | 0.01 | .990 |  | -287.97 (272.98) | -823.00 | 247.05 | -1.05 | .291 |
|  |  |  |  |  |  |  |  |  |  |  |  |  |  |
| Group (DCD) |  | | |  |  |  |  |  | 316.81 (404.96) | -476.90 | 1110.52 | 0.78 | .434 |
|  |  |  |  |  |  |  |  |  |  |  |  |  |  |
| Session 2 x Group (DCD) |  | | |  |  |  |  |  | 526.29 (493.12) | -440.20 | 1492.78 | 1.07 | .286 |
|  |  |  |  |  |  |  |  |  |  |  |  |  |  |
| Session 3 x Group (DCD) |  | | |  |  |  |  |  | 1021.98 (509.40) | 23.56 | 2020.39 | 2.01 | **.045** |
|  |  |  |  |  |  |  |  |  |  |  |  |  |  |
| **Random Effects** | | | | | | | | | | | | | |
| σ^2^ |  | | 3624048.95 | | | | |  | 3599692.23 | | | | |
| τ_00_ |  | | 506968.45 _subject_ | | | | |  | 423049.73 _subject_ | | | | |
| ICC |  | | 0.12 | | | | |  | 0.11 | | | | |
| N |  | | 33 _subject_ | | | | |  | 33 _subject_ | | | | |
| Observations |  | | 420 | | | | |  | 420 | | | | |
| Marginal R^2^ / Conditional R^2^ | |  | 0.000 / 0.123 | | | | |  | 0.039 / 0.140 | | | | |
| *Note.* Standard error presented in brackets; Comparison level for ‘Session’ = Session 1; Comparison level for ‘group’ = Control; DCD = Developmental Coordination Disorder. | | | | | | | | | | | | | |

**Figure 7**

*Individual Data Points for VI Efficiency for Individuals with DCD Across Sessions*


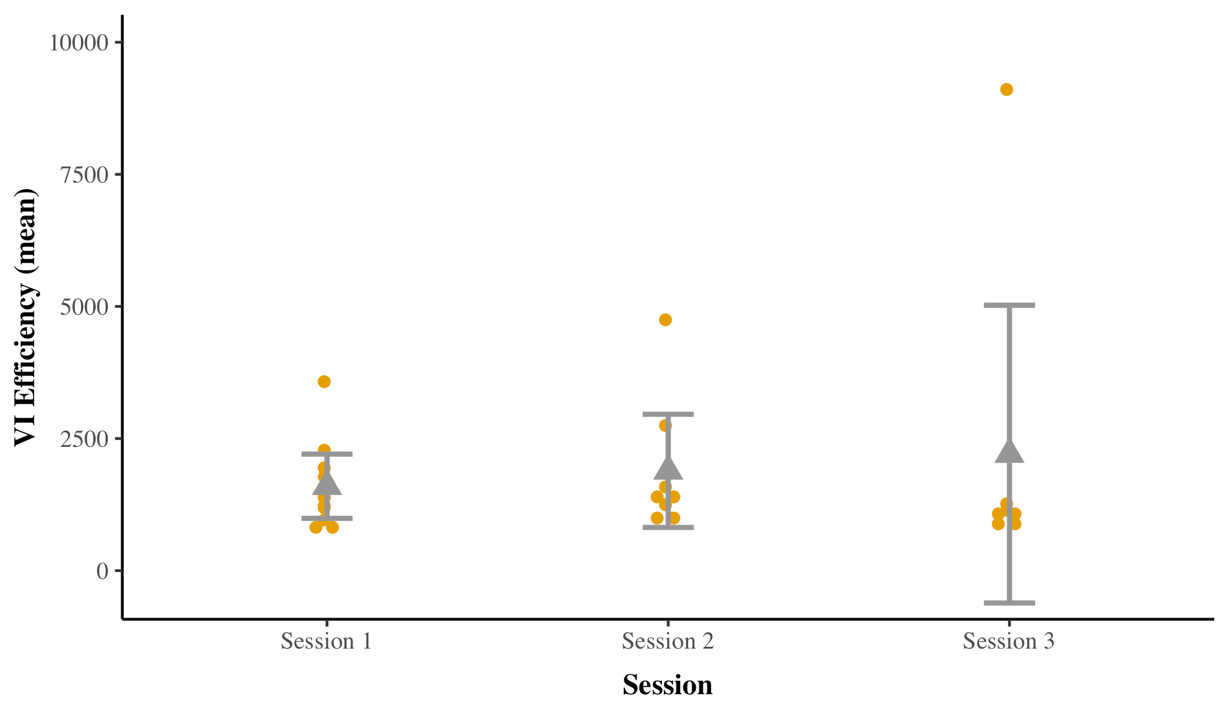


*Note.* Sample means and lower and upper 95% confidence intervals presented; VI = Visual Imagery; VI efficiency as indicated by letter number rotation task performance

**M**

| **Table 11**  *Linear Mixed Models for Motor Imagery Performance Efficiency Across Conditions* | | | | | | | | | | | |
| --- | --- | --- | --- | --- | --- | --- | --- | --- | --- | --- | --- |
|  | Model 1: Condition | | | | |  | Model 2: Condition x Group | | | | |
|  |  | 95% Confidence Interval | |  |  |  |  | 95% Confidence Interval | |  |  |
| *Predictors* | *Estimates* (*B*) | *Lower* | *Upper* | *t* | *p* |  | *Estimates* (*B*) | *Lower* | *Upper* | *t* | *p* |
| Intercept | 1277.37  (125.63) | 1031.13 | 1523.61 | 10.17 | **< .001** |  | 1154.55  (142.05) | 876.13 | 1432.97 | 8.13 | **< .001** |
| Active PMC | 49.43  (92.54) | -131.94 | 230.80 | 0.53 | .593 |  | 69.03  (111.32) | -149.15 | 287.22 | 0.62 | .535 |
| Active SMA | 104.08  (92.28) | -76.78 | 284.95 | 1.13 | .259 |  | -21.90  (111.32) | -240.09 | 196.29 | -0.20 | .844 |
| Active PMC x Group (DCD) |  |  |  |  |  |  | -66.30  (197.95) | -454.27 | 321.67 | -0.33 | .738 |
|  |  |  |  |  |  |  |  |  |  |  |  |
| Active SMA x Group (DCD) |  |  |  |  |  |  | 381.94  (196.60) | -3.39 | 767.26 | 1.94 | .052 |
|  |  |  |  |  |  |  |  |  |  |  |  |
| **Random Effects** | | | | | | | | | | | |
| σ^2^ | 480401.88 | | | | |  | 476705.16 | | | | |
| τ_00_ | 325634.80 _subject_ | | | | |  | 268785.36 _subject_ | | | | |
| ICC | 0.4 | | | | |  | 0.36 | | | | |
| N | 28 _subject_ | | | | |  | 28 _subject_ | | | | |
| Observations | 360 | | | | |  | 360 | | | | |
| Marginal R^2^ / Conditional R^2^ | 0.002 / 0.405 | | | | |  | 0.074 / 0.408 | | | | |
| *Note.* Standard error presented in brackets; Comparison level for ‘condition’ = Sham; Comparison level for ‘group’ = Control; PMC = Primary motor cortex; SMA = Supplementary motor area; DCD = Developmental Coordination Disorder. | | | | | | | | | | | |

| **Table 12**  *Linear Mixed Models for Visual Imagery Performance Efficiency Across Conditions* | | | | | | | | | | | | |
| --- | --- | --- | --- | --- | --- | --- | --- | --- | --- | --- | --- | --- |
|  | Model 1: Condition | | | | | |  | Model 2: Condition x Group | | | | |
|  |  | | 95% Confidence Interval | |  |  |  |  | 95% Confidence Interval | |  |  |
| *Predictors* | *Estimates* (*B*) | | *Lower* | *Upper* | *t* | *p* |  | *Estimates* (*B*) | *Lower* | *Upper* | *t* | *p* |
| Intercept | 1282.36  (201.45) | | 887.52 | 1677.20 | 6.37 | **< .001** |  | 1196.54  (231.98) | 741.88 | 1651.20 | 5.16 | **< .001** |
|  |  |  |  |  |  |  |  |  |  |  |  |  |
| Active PMC | 144.53  (232.84) | | -311.83 | 600.90 | 0.62 | .535 |  | -136.99  (273.70) | -673.44 | 399.46 | -0.50 | .617 |
|  |  |  |  |  |  |  |  |  |  |  |  |  |
| Active SMA | 86.72  (228.51) | | -361.16 | 534.59 | 0.38 | .704 |  | -80.99  (273.70) | -617.44 | 455.46 | -0.30 | .767 |
|  |  |  |  |  |  |  |  |  |  |  |  |  |
| Active PMC x Group (DCD) |  | |  |  |  |  |  | 1007.82  (514.68) | -0.94 | 2016.58 | 1.96 | .050 |
|  |  |  |  |  |  |  |  |  |  |  |  |  |
| Active SMA x Group (DCD) |  | |  |  |  |  |  | 511.72  (491.68) | -451.95 | 1475.39 | 1.04 | .298 |
|  |  |  |  |  |  |  |  |  |  |  |  |  |
| **Random Effects** | | | | | | | | | | | | |
| σ^2^ |  | 3619806.11 | | | | |  | 3599085.07 | | | | |
| τ_00_ |  | 513169.57 _subject_ | | | | |  | 424199.18 _subject_ | | | | |
| ICC |  | 0.12 | | | | |  | 0.11 | | | | |
| N |  | 33 _subject_ | | | | |  | 33 _subject_ | | | | |
| Observations |  | 420 | | | | |  | 420 | | | | |
| Marginal R^2^ / Conditional R^2^ |  | 0.001 / 0.125 | | | | |  | 0.038 / 0.140 | | | | |
| *Note.* Standard error presented in brackets; Comparison level for ‘condition’ = Sham; Comparison level for ‘group’ = Control; PMC = Primary motor cortex; SMA = Supplementary motor area; DCD = Developmental Coordination Disorder | | | | | | | | | | | | |

**N**

**Figure 8**

*
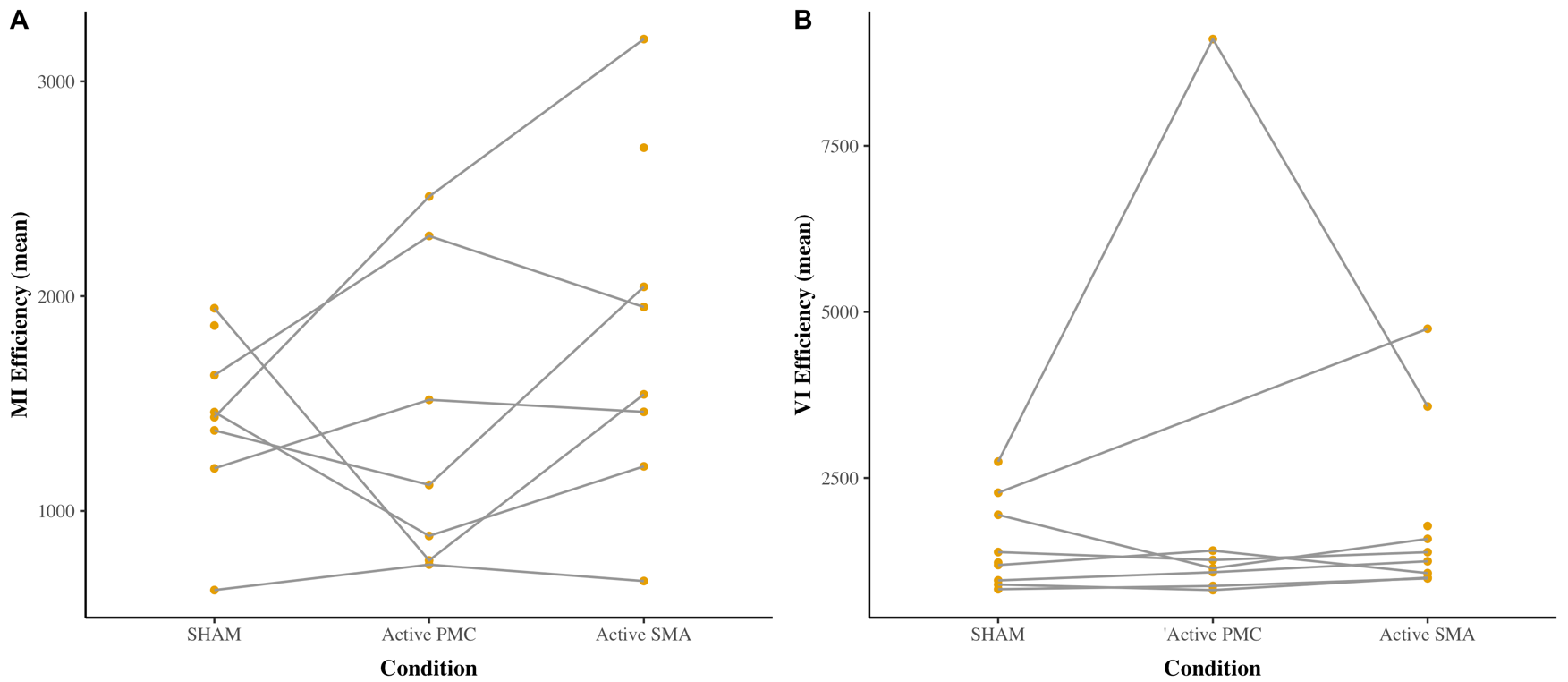
Individual Data Points for MI and VI Efficiency for Individuals with DCD Across Sessions*

DCD = Developmental Coordination Disorder; MI = Motor imagery; VI = Visual imagery; **A**. MI efficiency as indicated by performance on the hand rotation task; **B.** VI efficiency as indicated by performance on the letter number rotation task.

References

Bruyer, R., & Brysbaert, M. (2011). Combining speed and accuracy in cognitive psychology: Is the inverse efficiency score (IES) a better dependent variable than the mean reaction time (RT) and the percentage of errors (PE)? *Psychologica Belgica, 51*(1), 5-13. <https://doi.org/10.5334/pb-51-1-5>

Hoenig, J. M., & Heisey, D. M. (2001). The abuse of power: the pervasive fallacy of power calculations for data analysis. *The American Statistician, 55*(1), 19-24. <https://doi.org/10.1198/000313001300339897>

Perugini, M., Gallucci, M., & Costantini, G. (2018). A practical primer to power analysis for simple experimental designs. *International Review of Social Psychology, 31*(1). <https://doi.org/10.5334/irsp.181>

Townsend, J. T., & Ashby, F. G. (1978). Methods of modeling capacity in simple processing systems. *Cognitive theory, 3*, 199-139.

Townsend, J. T., & Ashby, F. G. (1983). *Stochastic modeling of elementary psychological processes*. CUP Archive.
